# Synthesis of potent vasodilating agents: in silico and in vitro evaluation of 6-(4-substitutedphenyl)-3-pyridazinone derivatives as potential hydralazine analogues

**DOI:** 10.1038/s41598-024-79697-1

**Published:** 2024-11-27

**Authors:** Marian W. Aziz, Khaled O. Mohamed, Doaa B. Farag, Amira Karam Khalifa, Zeinab Mahmoud

**Affiliations:** 1 Pharmaceutical chemistry department, Faculty of Pharmacy, Misr International University, Cairo, Egypt; 2 Pharmaceutical organic chemistry department, Faculty of Pharmacy, Cairo University, Cairo, Egypt; 3 Pharmaceutical chemistry department, Faculty of Pharmacy, Sinai University (Arish branch), Sinai, Egypt; 4 Medical pharmacology department, Faculty of Medicine, Cairo University, Cairo, Egypt; 5 Medical Pharmacology department, Faculty of Medicine, Nahda University, Beni Suef, Egypt

**Keywords:** Hypertension, Vasodilators, Pyridazinones, 3DQSAR pharmacophore, ADMET., Drug discovery, Chemical biology, Chemistry, Medicinal chemistry, Organic chemistry, Chemical synthesis

## Abstract

People of all age categories and lifestyles suffer to different extents from hypertension. Accordingly, this necessitates the rise of new ways to defeat this enemy. Vasodilators exert a principal portion of highly effectual antihypertensive agents; our research is focused on the design, synthesis and biological evaluation of a new series of 6-(4-substitutedphenyl)-3-pyridazinones as potential hydralazine vasodilator analogues implementing both in vitro and in silico approaches. All the synthesized compounds were assessed for their vitro vasorelaxant activity against multiple references. New members revealed potent vasorelaxant activity (EC50 = 0.02916–1.907 µM) compared to the conventional vasorelaxants hydralazine, diazoxide, isosorbitole mononitrate and nitroglycerin (EC50 = 18.21, 19.5, 30.1 and 0.1824 µM, respectively). Compounds 2e, 2h and 2j exerted superior activities compared to others with EC50 = 0.1162, 0.07154 and 0.02916 µM, respectively. The physiochemical properties and drug-likeliness behavior of the new derivatives were investigated by conducting ADMET studies.

**Supplementary Information:**

The online version contains supplementary material available at 10.1038/s41598-024-79697-1.

## Highlights

1. Vasodilators as one of the most important anti-hypertensive classes.

2. A new series of 6-(4-substitutedphenyl)-3-pyridazinones were designed and synthesized.

3. 3DQSAR pharmacophore model generation was conducted and used in activity prediction.

4. Compounds **2e**, **2h** and **2j** exerted superior in vitro vasorelaxant activities compared to hydralazine.

5. Most of the synthesized compounds revealed promising results in the in silico ADMET studies in accordance with the rational design.

**Supplementary Information:**

The online version contains supplementary material available at 10.1038/s41598-024-79697-1.

## Introduction

**Fig. 1:**
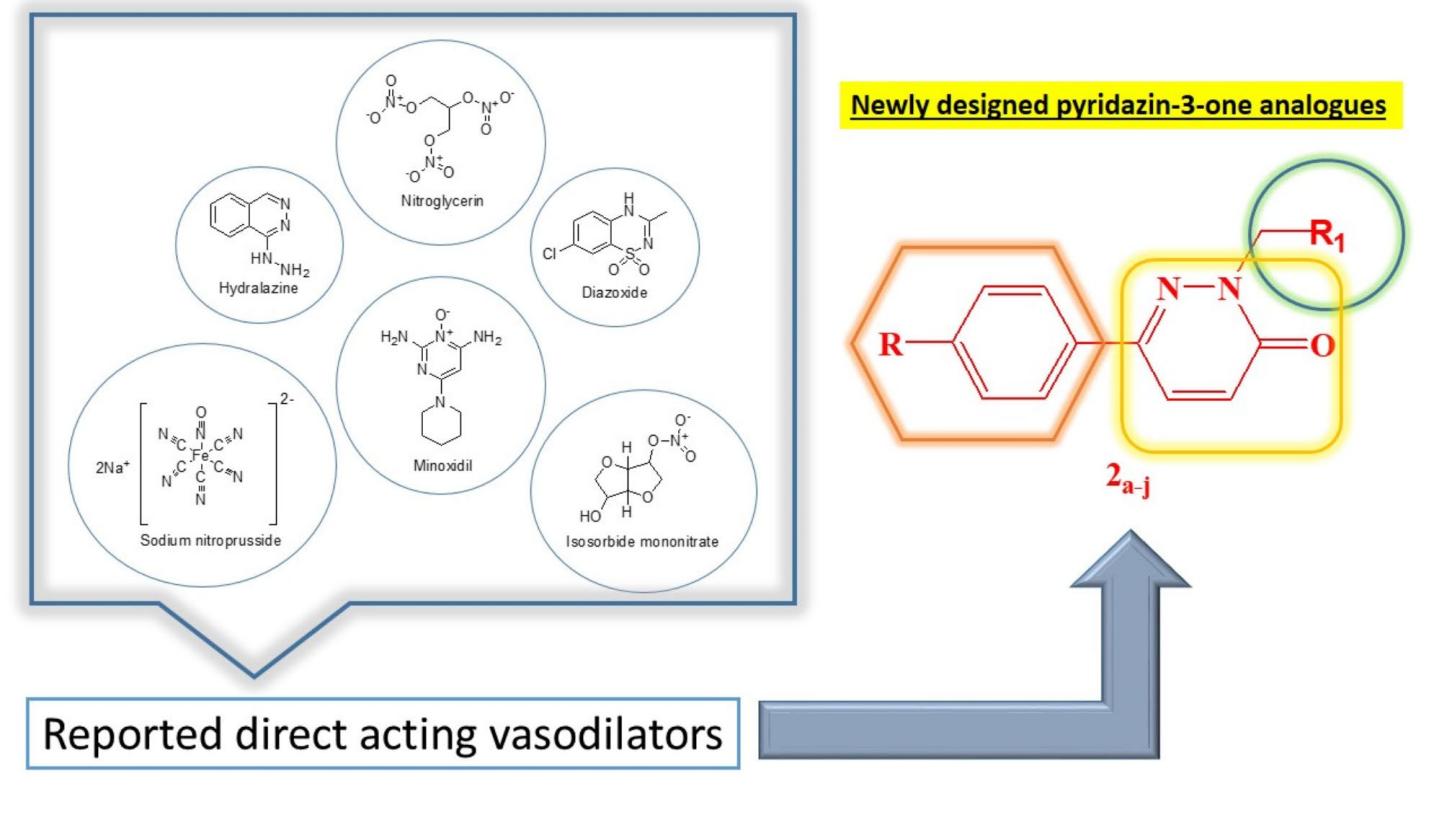
Various reported direct acting vasodilators along with the proposed design of newly synthesized pyridazin-3-one analogues.

Cardiovascular disorders (CVDs) enclose a wide range of heart and blood vessels disorders which encompass numerous high prevalence disorders as coronary artery disease, heart failure, stoke and most commonly expressed; hypertension. Such conditions significantly impact human’s health and quality of life as well as placing a heavy burden on health care systems^1^. According to World Health Organization (WHO), CVDs are the leading cause of death worldwide, accounting for approximately 32% of all global deaths in 2019, which translates to about 17.9 million deaths per year. Hypertension is commonly known as the silent killer, and is considered as one of the most pervasive cardiovascular chronic conditions that is characterized by elevated blood pressure in the arteries^2^. It’s diagnostic marker is having a blood pressure reading of 130/80 mmHg or higher, according to the American College of Cardiology (ACC) and the American Heart Association (AHA) guidelines^2–4^. Hypertension is classified into two main categories; a Primary (Essential) Hypertension: No identifiable cause, accounting for about 90–95% of cases and a Secondary Hypertension: Caused by underlying conditions such as kidney disease, hormonal disorders, or certain medications^5^. Considering its high prevalence and extreme health risks; the hypertension management is crucial in maintaining overall health and well-being^1^. The discovery of antihypertensive agents has been a continuous procedure starting with thiazide diuretics up to Aliskiren^®^ as the newest centrally active renin inhibitor discovered in 2000 ^6^. Unfortunately, due to this ailment complex pathophysiology, the ideal drug for it doesn’t really exist. However, it’s possible to map the ultimate feature for modern antihypertensive agents which have been of a great interest for several research groups^6^. Although several vasodilating agents are already available, such as angiotensin-converting enzyme inhibitors, angiotensin receptor blockers and calcium antagonists yet, these agents are frequently used in association with other drugs, since several patients become refractory to the therapy^7^. Moreover, none of the available agents are free of side effects which applies too for direct acting vasodilators that work by directly relaxing the smooth muscles of blood vessels accordingly, leading to vasodilation by reducing vascular resistance and consequently lowering the blood pressure^8,9^. This emphasizes the need of searching for novel vasodilating agents^7^ as inspired by hydralazine; being the first discovered direct acting vasodilator in the 1950s and primary used for the treatment of hypertension. Since its discovery marked a significant advancement in the pharmacological management of hypertension in terms of providing an effective option for patients, especially those who did not respond well to other antihypertensive medications^10,11^. Our research proposal is based on designing and synthesizing new hydralazine analogues with pyridazin-3-one moiety as a core structure instead of the fused phthalazine (benzopyridazine) ring found in hydralazine structure (Fig. 1) due to the garnered interest in pyridazinone based compounds due to their remarkable diverse biological activities recorded in literature^12^. Recently, pyridazinone derivatives are proven to be promising in managing high blood pressure. Their structure allows for the modification of various functional groups, which can lead to a wide range of effects. This versatility makes them valuable in medicinal chemistry^12^. Furthermore, we studied the addition of different substitutions at position 2 of the pyridazinone ring using a methyl linker as well as investigating a number of positions as in 6 substitutions to illustrate the effect of such substituents on the observed activity. A comprehensive comparison between the suggested structural changes conducted in our derivatives on the vasodilatory activity against the reference (hydralazine) as well as other direct acting vasodilators that was achieved through both 3D-QSAR pharmacophore modeling protocol evaluation and a detailed rat aortic vasodilation of serial dilution of the proposed analogues to deduct EC_50_ values compared to those of references.

## Results and discussion

### Chemistry

**Scheme 1:**
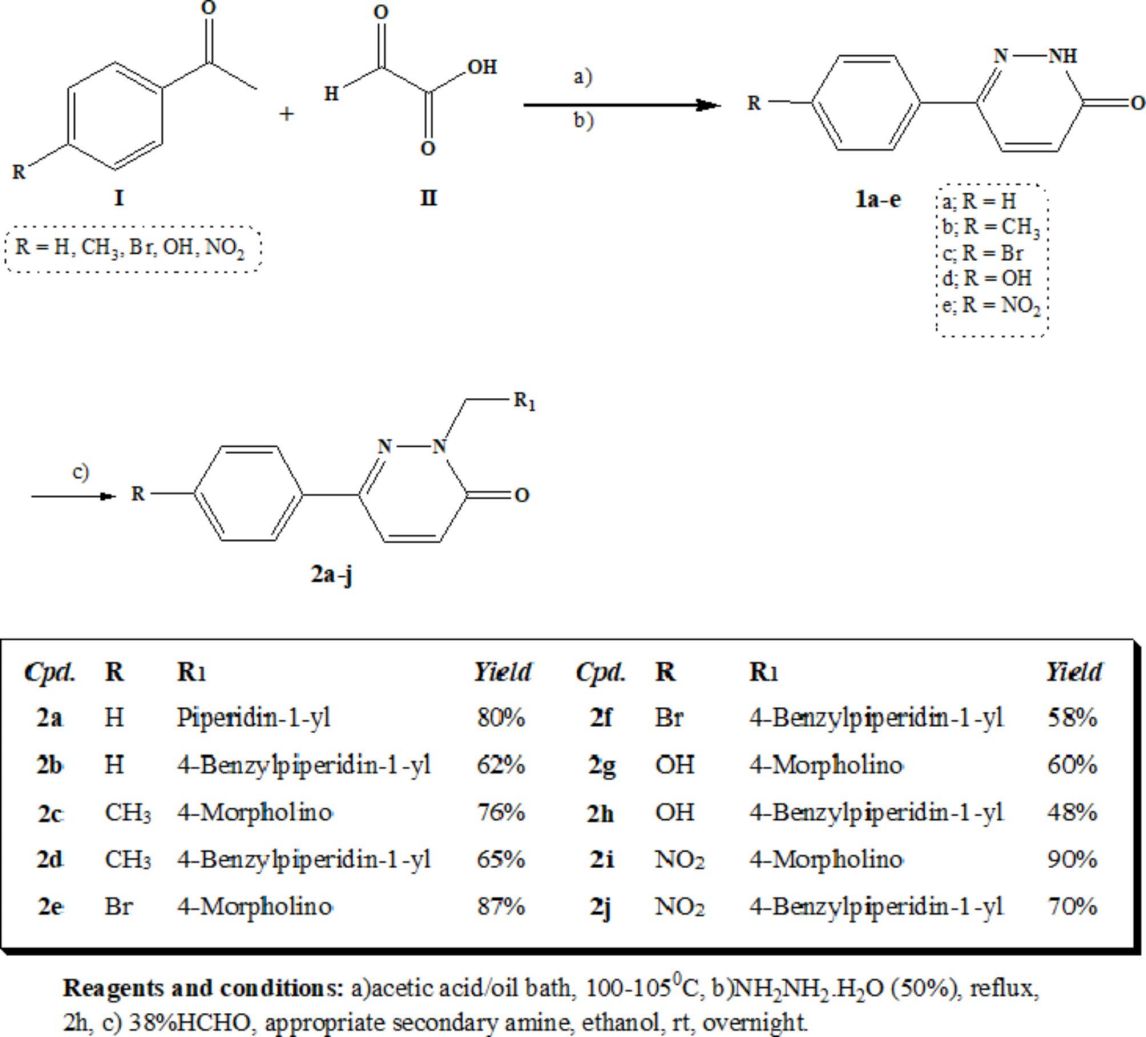
Preparation of compounds **2a-j**.

The synthetic pathways utilized in preparing the target compounds are discussed in scheme 1. Scheme 1 utilizes the in-situ synthesis of pyridazin-3-one derivatives **2a-j** by the reaction of different *p*-substituted acetophenone **I** with glyoxylic acid **II**^13^. Cyclization with hydrazine hydrate afforded the intermediates **1a-e**. The previous step was conducted as a multistep fusion reaction between glyoxylic acid **II** and liquid substituted acetophenone **I** in an oil bath. However, acetic acid was adapted as a solvent for substituted acetophenone **I** that existed in a solid state condition^14,15^. In both cases, the resulting mixtures were then diluted with water and their pH was adjusted to 8 using ammonium hydroxide aqueous solution (25%). The mixture was subsequently filtrated from any residues and purified from any impurities by extracting with methylene chloride. The separated aqueous layer was then refluxed with hydrazine hydrate (50%) for 2h to yield the targeted intermediate **1a-e** upon cooling. Refluxing the different intermediates **1a-e** with the appropriate cyclic secondary amines in presence of formalin aqueous solution (38%) in absolute ethanol as a solvent afforded the target compounds **2a-j**. The structure of compounds **2a-j** were depicted by spectral data. The disappearance of NH group peak of at the range of 3468–3251 cm^-1^ in the IR spectra, in addition to the development of the C = O groups peak of the pyridazinone ring found at 1681–1651 cm^-1^ in the newly synthesized compounds spectra were two clear clues for the success of the reaction. The ^1^H NMR revealed the disappearance of signals at 11.11–12.59 ppm of the NH moiety along with the disappearance of the methyl group’s protons of the reactant substituted acetophenone and the confirmed appearance of extra aromatic protons at 6.83–8.34 ppm and the development of peak of the pyridazinone C = O carbon at the range of 159.1–160.7 ppm in the ^13^C NMR.

### Molecular modeling studies

Computer-aided drug design (CADD) is the science of utilizing computers to enhance commercially available drugs that are used as lead compounds to design new ligands that are potentially active against certain biological targets^16^. Quantum mechanics and molecular modelling techniques are deemed the core science for CADD. CADD helps to limit the options of infinite numbers in lead optimization process which is superior to traditional ways for discovering new drugs that consume huge amounts of money, time and effort since it predicts the promising drug compounds in order to be synthesized^16,17^.

In this present study, we synthesized number of pyridazin-3-one derivatives as novel vasodilating agents in terms of predicting their promising activity prior their biological evaluation by using indirect CADD molecular modeling techniques. First, we studied previously reported bioactive compounds related to our desired activity, and then we generated 10 predictive pharmacophore models elucidating the 3D quantitative relationship between structure and activity of a set of 22 compounds having reported activity as vasodilators by using 3DQSAR pharmacophore generation protocol. The pharmacophores generated were then validated to select the best model to map our proposed compounds for the evaluation of their fitting to the model features as well as prediction of their estimated activity.

#### 3DQSAR pharmacophore model generation and validation

For the generation of Vasodilators library, a set of 22 vasodilating molecules with known activity (EC_50_) ranging from 0.339 to 114.3 µM were collected from literature^10,18^. The ligand library was prepared by using Accelrys Discovery Studio 4.0 software to carry out protonation and minimize energy as well as fixing bad valences and generating 3D coordinates. For 3DQSAR pharmacophore model generation Accelrys Discovery Studio 4.0 software was used to create a ligand-based pharmacophore model. The collected ligand library was divided into a training set of 13 Ligands and a test set of 9 ligands. For developing the protocol, hydrogen bond acceptors (HBAs), hydrogen bond donor (HBDs), hydrophobic moieties (HYPs), positive ionizable groups (PIs) and aromatic rings (ARs) were selected as the chemical features based on the result of a conducted feature mapping protocol for the training set ligands. The uncertainty was set to 1.5 and EC_50_ was selected as the activity measure. The generated pharmacophore models (10 hypothesis) were then validated based on its statistical significance and its ability to predict the biological activity of unknown ligands, cost difference and accuracy of estimated activity as well as the reference mapping results whereas, the validity of the model can be proved.

Hypogen algorithm generates multiple hypothesis (10 hypotheses as default) and the best one is selected based on the cost difference. The total cost of each hypothesis is the result of the summation of weight, error and configuration cost, the minimum possible cost for a model that perfectly fit is called the fixed cost and the maximum possible cost for a model that has no features, all of those are calculated for this algorithm as well.

$$Cost{\text{ }}difference = \left( {Null{\text{ }}cost - fixed{\text{ }}cost} \right)-\left( {Total{\text{ }}cost - fixed{\text{ }}cost} \right).$$

For considering the model to be statistically significant, the cost difference must be greater than 40 where the hypothesis of the highest cost difference will be selected to carry on further investigations, mapping of the reference ligand Hydralazine is carried on as a further validation for the pharmacophore model and to use it to compare its fit value to that of the proposed analogues.

**Fig. 2:**
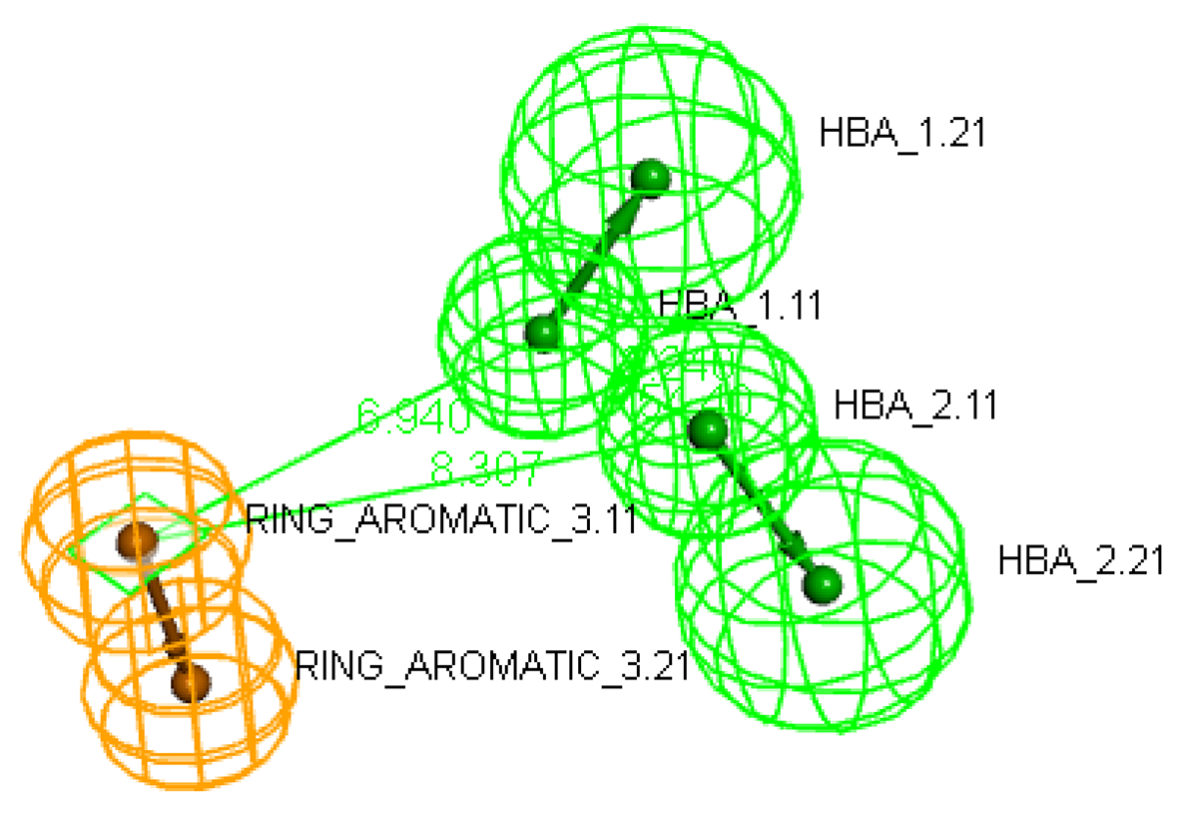
The best generated pharmacophore model hypothesis considering constraint distances and angles between its location features in which hydrogen bond donor (HBA) features are colored in green while ring aromatic (RA) feature is colored in orange.

The analysis of the 10 hypotheses revealed that the best was the first model. This model showed the highest cost difference of 62.030 indicating statistical significance of the model with high predictive power > 90%. Its RMS value was 2.39 with correlation coefficient of 0.813 revealing the suitability of the model to predict novel compounds activity as shown in Table 1. The valid model showed 2 hydrogen bond acceptors and 1 ring aromatic features (Fig. 2).

**Table 1:** The ten generated hypotheses with their maximum fit, total cost, cost difference, RMS and correlation coefficient.

Hypothesis	Maximum fit	Total cost	Cost difference	RMS	Correlation coefficient
**1**	6.699	78.203	62.030	2.390	0.813
**2**	7.330	87.920	52.312	2.662	0.761
**3**	5.439	91.703	48.530	2.793	0.732
**4**	4.646	105.191	35.042	3.12	0.649
**5**	4.916	106.755	33.478	3.168	0.635
**6**	4.275	110.204	30.029	3.225	0.619
**7**	5.166	111.228	29.005	3.248	0.614
**8**	3.937	111.301	28.932	3.232	0.616
**9**	4.581	112.050	28.182	3.282	0.600
**10**	4.127	121.294	18.939	3.472	0.534

**Fig. 3:**
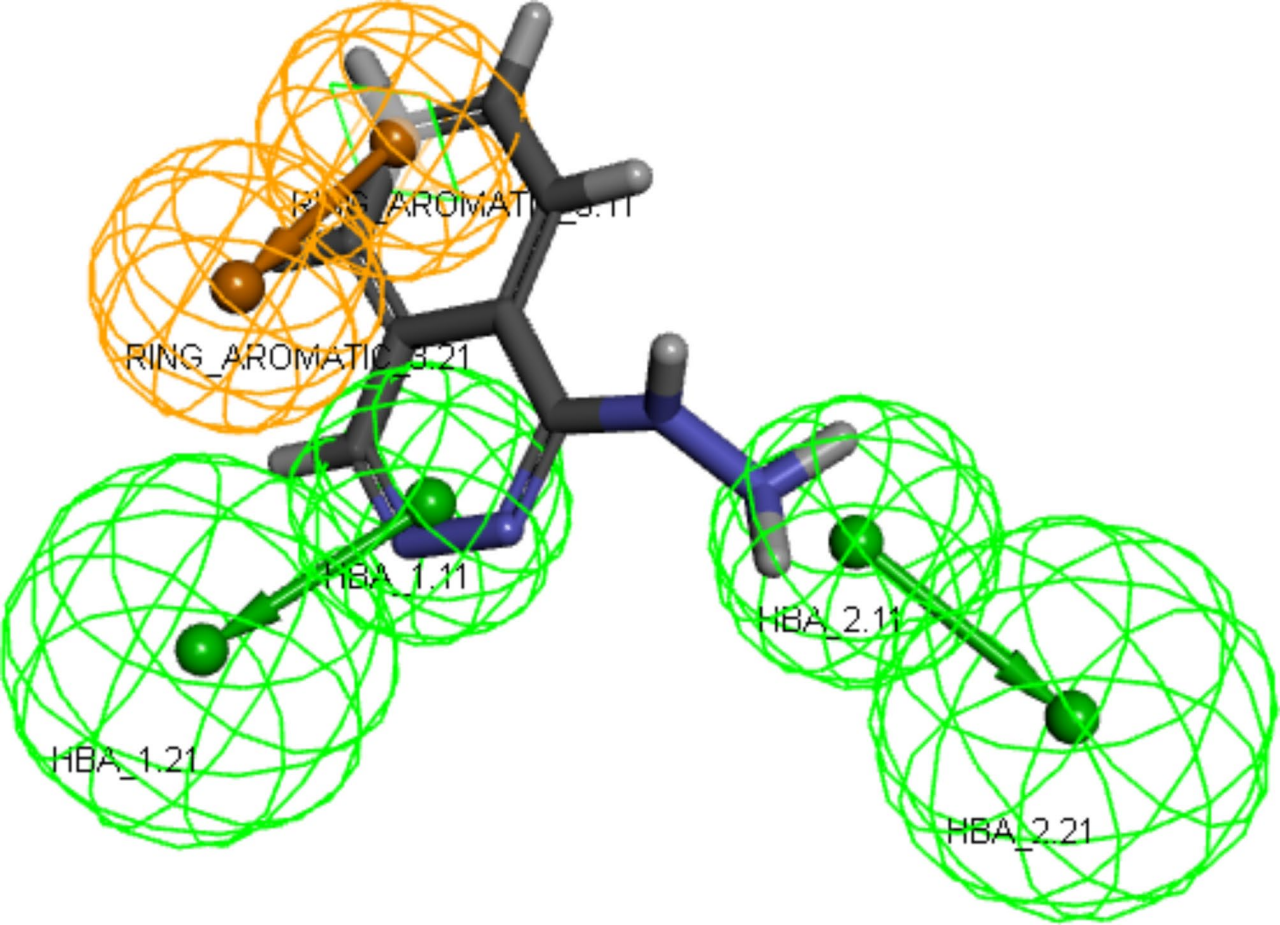
The reference ligand (hydralazine) mapped inside the hit hypothesis constraints with fit value of 4.19945 and estimated EC_50_ of 30.546 µM.

Mapping of hydralazine into the valid model was carried out using ligand-pharmacophore mapping protocol of Accelrys Discovery Studio 4.0 software which revealed fit value of 4.19945 and estimated EC_50_ of 30.546 µM (Fig. 3).

#### Pharmacophore mapping of the proposed compounds

Pharmacophore mapping of the proposed compounds **2a-j** was carried out by using the same ligand-pharmacophore mapping protocol used for the reference hydralazine to determine their fit value and estimated EC_50_ (Table 2). The determined fit values showed their compatibility with the pharmacophore model. Furthermore, all the proposed analogues were observed to have promising estimated EC_50_ values compared to the reference hydralazine as well as other reported ligands used in building the valid model.

**Table 2:** Mapping results of the proposed vasodilators, compounds **2a-j**, to the generated valid pharmacophore model.

Compounds	Fit value	Estimated activity (µM)
2a	5.38778	10.0398
2b	5.22513	2.87921
2c	6.3134	0.234964
2d	6.48242	0.159214
2e	6.33229	0.224963
2f	5.80112	0.764334
2g	6.31648	0.233302
2h	5.46622	1.65265
2i	6.33486	0.223631
2j	6.41419	0.186297

### Biological evaluation

Newly synthesized compounds **2a-j** along with their standard reference that went through 3D QSAR pharmacophore molecular modelling protocol verifying their scientific potentials were then screened for their vasodilatory activity in vitro on thoracic aorta dissected out from Healthy 50 Wistar Albino rats that is pre-contacted using submaximal concentration of phenylephrine to elucidate the relaxant response of different prepared concentration of these targeted drugs in order to calculate valid EC_50_ values for each.

#### Vasorelaxant activity

**Fig. 4:**
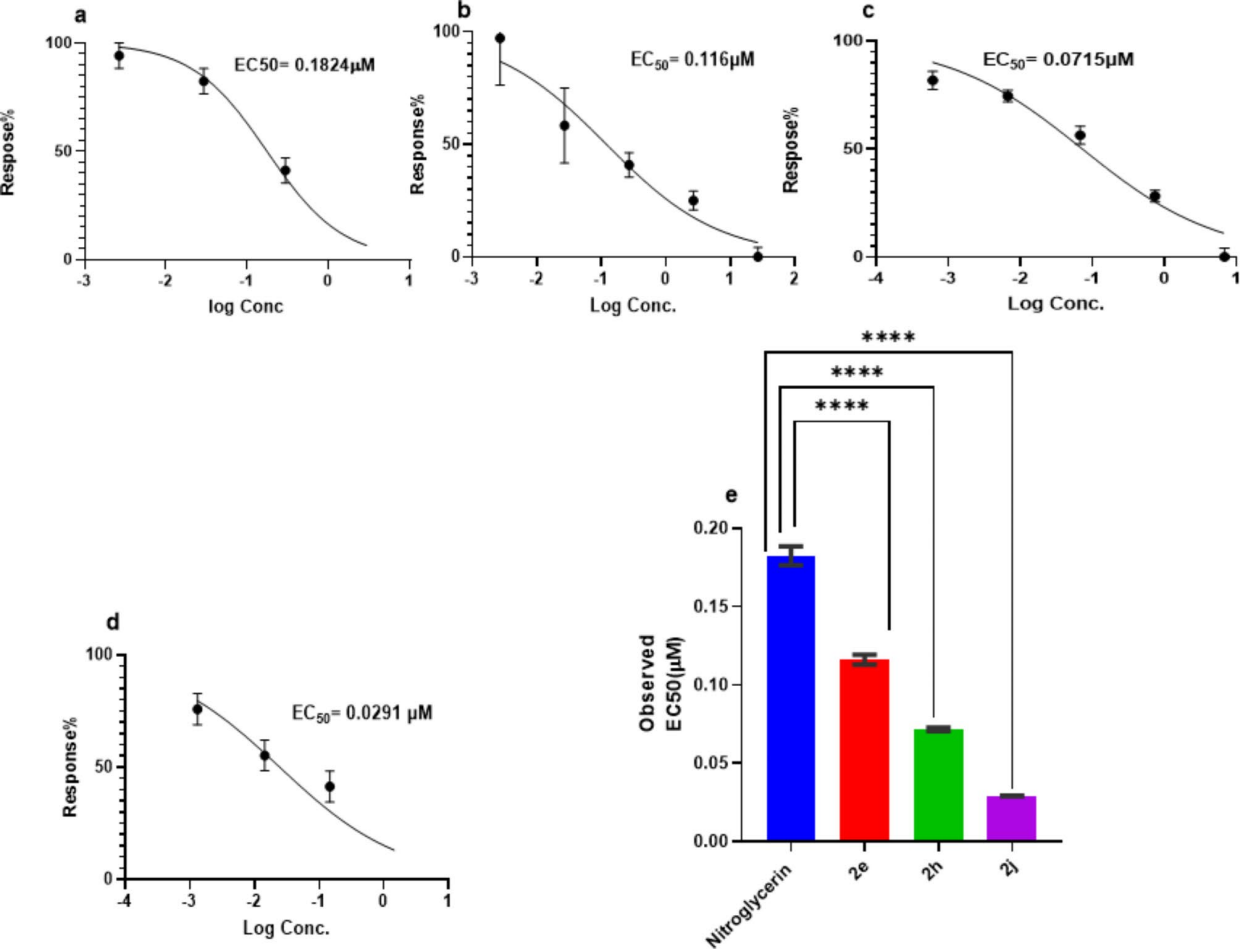
Dose response curve with the mean EC50 for compounds; **(a)**: Nitroglycerin, **(b)**:2e compound **(c)**: 2h, **(d)**: 2j and **(e)**: Mean EC50. Values represent means ± SD (*n* = 5rats for each investigated drug). A significant difference is reported when *P* is less than 0.05 and determined by one -way ANOVA followed by pairwise comparison using Dunnet ’s test. ****statistically significant as compared to the nitroglycerin control group (*P*˂0.0001).

**Fig. 5:**
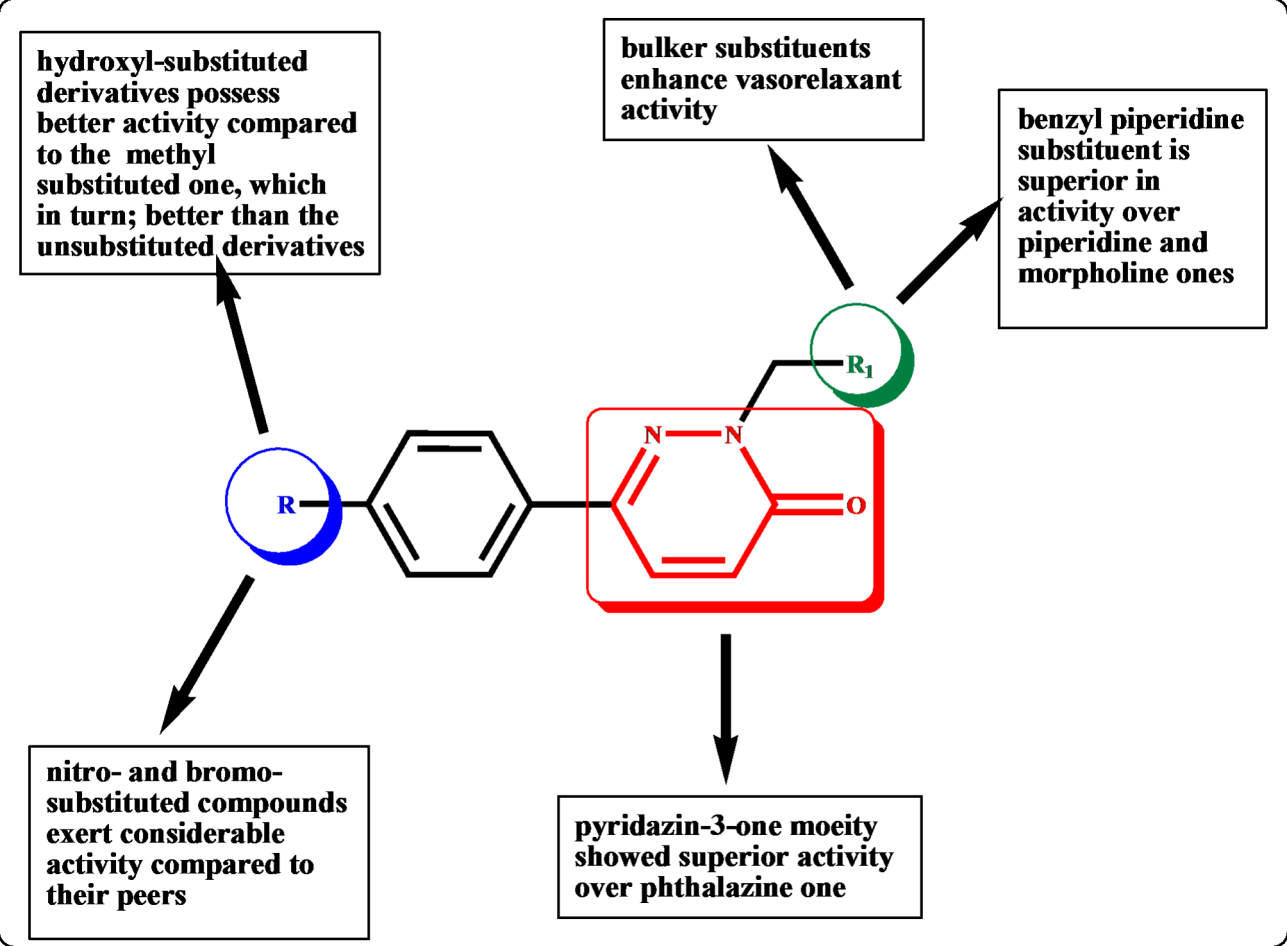
The structure activity relationship for the newly synthesized pyridazin-3-one derivatives.

Compounds **2a-j** were inspected for their vasorelaxant activity on thoracic aorta of Wistar Albino rats according to the reported procedure^19^. Hydralazine, diazoxide, isosorbide mononitrate and nitroglycerin were used as reference standards. The resulting data was presented in Table 3 as effective drug concentration causing 50% relaxation of the norepinephrine contraction of mice aorta rings (EC_50_) in (µM). From the obtained results, it can be noted that all the tested compounds emphasized potent range of activity with EC_50_ values of 0.02916–1.907 µM compared to hydralazine, isosorbide mononitrate, diazoxide and nitroglycerin EC50 of 18.210, 30.1, 19.5 and 0.1824, respectively. Compounds **2e**, **2h** and **2j** showed superb vasorelaxant activity compared to hydralazine (the used lead compound for structural based design) with EC_50_ of 0.1162, 0.07154 and 0.02916 µM, respectively (Fig. 4). The observed structure activity relationship revealed that the pyridazine-3-one in compounds **2a-j** instead of the phthalazine ring of the hydralazine; increased the activity tremendously. The different substituents of the phenyl ring at position 6 of the pyridazinone affected the activity of compounds. Compounds **2h** and **2g** with hydroxyl group showed superior activity of EC_50_ = 0.07154 and 0.2180 µM followed by compounds **2c** and **2d** having methyl group with EC_50_ = 0.2624 and 0.1916 µM and then came those of the unsubstituted phenyl (**2b** and **2a**) with EC_50_ = 0.2250 and 0.4240 µM. However, only one of the nitro-substituted derivatives (**2j**) and the bromo-substituted derivatives (**2e**) showed highly remarkable activity compared to their peers with EC_50_ = 0.02916 and 0.1162 µM respectively. Regarding the cyclic secondary amine in position 2 of the pyridazinone backbone; bulker substituent such as 4-benzylpiperidine moiety showed higher activity as in compound **2j** (EC_50_ = 0.02916 µM) than less bulker ones such as piperidine and morpholine as in compounds **2a** (EC_50_ = 0.4240 µM) and **2g** (EC_50_ = 0.2180 µM) (Fig. 5).

**Table 3:** Vasorelaxant activity (EC_50_, µM and emax, %) of the tested compounds.

Compound no.	EC_50_ (µM)^a^	Emax ± SD (%)	Compound no.	EC_50_ (µM)^a^	Emax ± SD (%)
**2a**	0.42 ± 0.02	90.94 ± 0.70	**2f**	1.899 ± 0.003271	93.70 ± 0.28
**2b**	0.22 ± 0.01	91.24 ± 0.24	**2g**	0.2180 ± 0.001581	96.98 ± 0.32
**2c**	0.26 ± 0.006	97.18 ± 0.59	**2h**	0.07154 ± 0.00113	99.81 ± 0.73
**2d**	0.19 ± 0.004	97.51 ± 0.43	**2i**	1.907 ± 0.00507	98.26 ± 0.27
**2e**	0.11 ± 0.003	95.79 ± 0.24	**2j**	0.02916 ± 0.0002702	99.74 ± 0.19
**Hydralazine**	18.21^b^	**-**	**Diazoxide**	19.5^b^	-
**Isosorbide mononitrate**	30.10^b^	**-**	**Nitroglycerin**	0.1824 ± 0.005983	95.58 ± 1.96

^a^ EC_50_: Effective drug concentration causing 50% relaxation of the phenylephrine contraction of rat aorta rings. ^b^ According to the reported results^10,20,21^

*Value represents the mean ± SD, *n* = 5.

### ADME and toxicity studies

**Fig. 6:**
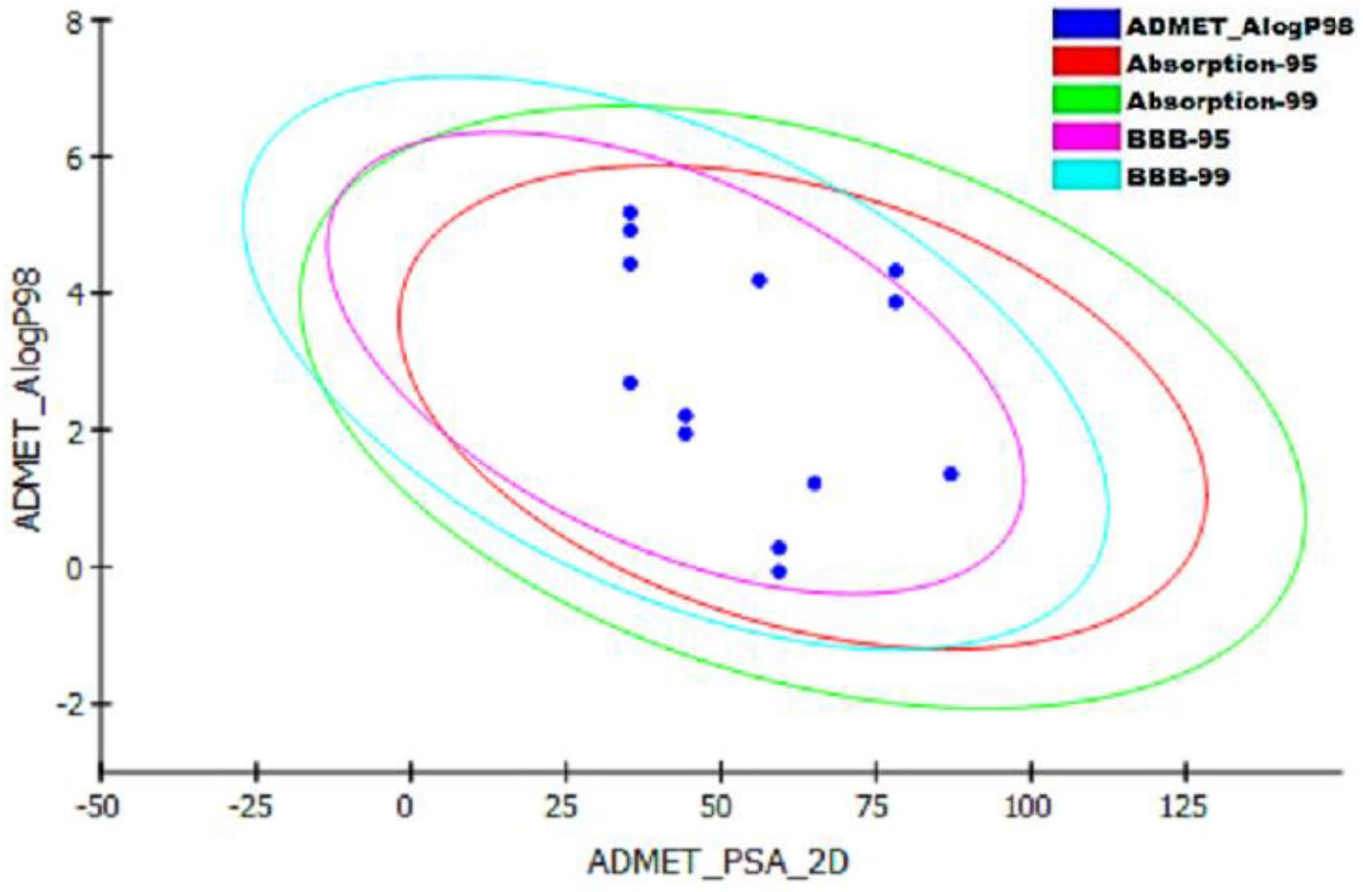
ADMET Description plot illustrating the human intestinal absorption (HIA) and blood brain barrier penetration prediction for the newly synthesized compounds.

When results were compared to the reference level values obtained from the Accelrys Discovery Studio 4.5, all compounds had optimal aqueous solubility and better druglike properties except compound **2f** that showed no but possible aqueous solubility. Among the studied ligands, compounds **2c**, **2e**, **2g**, **2i**, **2j** showed no Blood Brain Barrier penetration (Fig. 6). All compounds appeared to exert good intestinal absorption and illustrated different levels of plasma protein binding. Furthermore, the compounds were non-hepatotoxic except compounds **2e**, **2g**, **2i**, **2j**. Compounds **2c**, **2g**, **2i**, **2j** were cytochrome *P450* enzyme non-inhibitors as shown in Table 4.

According to the in silico toxicity studies illustrated in Table 5, it was found that none of the ligands showed mutagenicity except compound **2i**, all compounds were non-carcinogenic in mouse and rat models. Moreover, only three compounds were biodegradable in aerobic conditions.

**Table 4:** In silico ADMET screening of the synthesized compounds **2a-j**.

Compound	Aqueous Solubility Level^1^	Blood Brain Barrier level^2^	Human Intestinal Absorption Level^3^	Hepatotoxicity prediction^4^	CYP2D6 prediction^5^	Plasma Protein Binding prediction^6^
2a	3	1	0	false	true	true
2b	2	1	0	false	true	true
2c	3	2	0	false	false	false
2d	2	0	0	false	true	true
2e	3	2	0	true	true	false
2f	1	0	0	false	true	true
2g	3	3	0	true	false	false
2h	2	1	0	false	true	true
2i	3	3	0	true	false	false
2j	2	2	0	true	false	true

^1^ (1) No, but possible, (2) Yes, low, (3) Yes, well.

^2^ (0) Very high, (1) High, (2) Medium, (3) Low.

^3^ (0) Good.

^4^ (false) Non-hepatotoxic, (true) Hepatotoxic.

^5^ (false) Non inhibitor, (true) inhibitor.

^6^ (false) not bound to plasma protein, (true) bound to plasma protein.

**Table 5:** Toxicity profile of the synthesized compounds **2a-j** using toxicity prediction, extensible protocol of Accelrys Discovery studio 4.5.

Compound	Aerobic Bio-Degradability	Ames Prediction	Carcinogenicity Potency in mouse	Carcinogenic Potency in rat	TOPKAT_Rat Oral LD_50_ (g/kg body weight)	TOPKAT_Rat Maximum Tolerated Dose_Feed (g/kg body weight)	TOPKAT_Rat Maximum Tolerated Dose_Gavage (g/kg body weight)
2a	Degradable	Non-Mutagen	NO	NO	0.704822	0.0811332	0.0818904
2b	Non-Degradable	Non-Mutagen	NO	NO	0.447766	0.083154	0.0685762
2c	Degradable	Non-Mutagen	NO	NO	6.1736	0.0429369	0.0838771
2d	Non-Degradable	Non-Mutagen	NO	NO	0.838456	0.0687107	0.0526526
2e	Non-Degradable	Non-Mutagen	NO	NO	2.13901	0.0444935	0.00920672
2f	Non-Degradable	Non-Mutagen	NO	NO	0.277806	0.0680889	0.00552672
2g	Degradable	Non-Mutagen	NO	NO	1.55518	0.173071	0.0428836
2h	Non-Degradable	Non-Mutagen	NO	NO	0.222574	0.276438	0.0268914
2i	Non-Degradable	Mutagen	NO	NO	4.71945	0.039854	0.0344951
2j	Non-Degradable	Non-Mutagen	NO	NO	0.665194	0.0623034	0.0211534

## Conclusion

A series of ten 2,6-disubstituted-pyridazin-3-one derivatives 2a-j were synthesized and evaluated for their in-vitro vasorelaxant activity on thoracic aorta. All synthesized compounds revealed potent activity (EC50 = 0.02916–1.907 µM) compared to conventional vasorelaxants such as hydralazine, diazoxide, isosorbitole mononitrate and nitroglycerin (EC50 = 18.21, 19.5, 30.1 and 0.1824 µM, respectively). Compounds 2e, 2h and 2j exerted superior activity compared to others with EC50 = 0.1162, 0.07154 and 0.02916 µM, respectively. The ADME and toxicity parameters for the newly prepared series revealed that most of them exhibited good oral bioavailability, low to moderate blood brain barrier (BBB) penetration, very good intestinal absorption and minimal hepatotoxicity. The toxicity profiling of those compounds showed non-mutagenic and non-carcinogenic potencies. It can be deducted that 2-substituted-6 (4-subsitutedphenyl) pyridazin-3-one can be considered for further in-vivo evaluation and implemented as a base for additional optimization to acquire new promising vasorelaxant candidates.

## Experimental

### Chemistry

Melting points were obtained using BUCHI B-540 apparatus on open glass capillaries, faculty of Pharmacy, Misr International University and were uncorrected. IR spectra were recorded using KBr disks on Shimadzu IR 435 spectrophotometer (Shimadzu Corp., Kyoto, Japan), Faculty of Pharmacy, Cairo University, Cairo, Egypt and values were represented in cm^-1^^1^. H NMR spectra were carried out in DMSO-d_6_ with Bruker Avance-400 spectrophotometer operating at 400 MHz and 100 MHz (Bruker Crop., Billerica, MA, USA) spectrophotometer, Faculty of Pharmacy, Cairo University, Cairo, Egypt and at special unit facility, Faculty of Pharmacy, Ain-Shams university, Cairo, Egypt. Chemical Shifts were recorded in ppm δ scale, peak multiplicities are designed as follows: s, singlet; d, doublet; t, triplet; m, multiples^13^. C NMR spectra were carried out on Bruker 100 MHz spectrophotometer, Faculty of Pharmacy, Cairo University, Cairo, Egypt. Microanalysis for C, H and N were carried out at the Regional Center of Mycology and Biotechnology, Faculty of Pharmacy, Al-Azhar University using the Elemental Vario El Germany Instrument. The HR-MS was performed in the Natural Products Research Lab, Faculty of Pharmacy, Fayoum University. The progress of the reactions was monitored by TLC and performed on 0.255 mm pre-coated aluminum sheet silica gel MERCK 60 F 254 and was visualized by a UV lamp (254 nm) in an appropriate mobile phase mixture of hexane, methylene chloride and methanol.

#### Compounds **1a-e** were prepared as the reported methods with the following modifications^13–15,22–26^

In an oil bath, 0.05 mol (3.7 g) glyoxalic acid and 0.15 mol appropriate acetophenone derivatives were heated for 2h at 100–105 ^0^C. The reaction mixture was then cooled down to 40 ^º^C, the medium pH was adjusted to 8 using 20 ml water and 5 ml ammonium hydroxide solution (25%). After adjusting the pH, the reaction mixture was extracted with methylene chloride (4 × 25 ml). 0.05 mol (2.5 ml) hydrazine hydrate was added to the aqueous layer and the reaction mixture was refluxed again for 2h then allowed to cool down to room temperature. The resulting precipitate was filtered and recrystallized from ethanol.

#### ***General procedure for the preparation of compounds*** (**2a-j**)

An equimolar mixture of the appropriate secondary amine and the appropriate member from series **1** in absolute ethanol (10 ml). Formalin solution (38%, 0.3 ml) was then added to the mixture and was stirred overnight at room temperature. The reaction mixture was poured over ice cold water and the resulting precipitate was filtered. This solid was washed twice with water and recrystallized from methanol to yield compounds 2a-j.

##### **6-Phenyl-2-(piperidin-1-ylmethyl) pyridazin-3(2*H*)-one** (**2a**)

Yield: 80%, m.p. 122–124 °C; IR (KBr), cm^-1^: 3047 (arom.CH), 2866 (aliph.CH), 1651 (C = O);^1^H NMR (400 MHz, DMSO-d_6_) δ (ppm): 1.30–1.33 (m, 2h, CH_2_, C4 of piperidine), 1.45–1.50 (m, 4 H, 2CH_2_, C3 & C5 of piperidine), 2.64 (t, *J* = 8 Hz, 4 H, C2 & C6 of piperidine), 4.99 (s, 2h, methylene CH_2_), 7.05 (d, *J* = 8 Hz, 1 H, ArH of C5 pyridazinone), 7.05 (d,, *J* = 8 Hz, 1 H, ArH of C4 pyridazinone), 7.50 (d, *J* = 8 Hz, 2h, ArH), 7.85–7.88 (m, 2h, ArH), 8.03–8.06 (m, 1 H, ArH);^13^C NMR (100 MHz, DMSO-d6) δ (ppm): 23.9 (C-4 piperidinyl), 26.1 (C-3 & C-5 piperidinyl), 51.6 (C-2 & C-6 piperidinyl), 72.3 (CH_2_), 126.1 (C-4 pyridazinone), 129.7 (C-3 & C-5 phenyl), 130.5 (C-2 & C-6 phenyl), 131.9 (C-4 phenyl), 134.8 (C-1 phenyl), 142.9 (C-5 pyridazinone), 144.3 (C-6 pyridazinone), 160.4 (C = O pyridazinone). Calcd for C_16_H_19_N_3_O (269.34): C, 71.35; H, 7.11; N, 15.60: Found: C, 71.53; H, 7.20; N, 15.84%.

##### **2-((4-Benzylpiperidin-1-yl) methyl)-6-phenylpyridazin-3(2*H*)-one** (**2b**)

Yield: 62%, m.p. 103–105 °C; IR (KBr), cm^-1^: 3047 (arom.CH), 2866 (aliph.CH), 1651 (C = O), 1462;^1^H NMR (400 MHz, DMSO-d_6_) δ (ppm): 1.08–1.20 (m, 2h, CH_2_, C3 of piperidine), 1.30–1.52 (m, 2h, CH_2_, C5 of piperidine), 1.75 (t, *J* = 12hz, 2h, CH_2,_ C2 of piperidine), 2.24 (t, *J* = 12hz, 1 H, CH, C4 of piperidine), 2.44–2.50 (m, 2h, CH_2_, C6 of piperidine), 2.87 (d, *J* = 12hz, 2h, benzyl CH_2_), 5.00 (s, 2h, methylene CH_2_), 7.04 (d, *J* = 8 Hz, 1 H, ArH of C5 of pyridazinone), 7.11 (t, *J* = 12hz, 1 H, ArH), 7.16 (t, *J* = 16 Hz, 2h, ArH), 7.21–7.28 (m, 2h, ArH), 7.44–7.51 (m, 3 H, ArH), 7.87 (d, *J* = 4 Hz, 2h, ArH), 8.03 (d, *J* = 8 Hz, 1 H, ArH of C4 of pyridazinone);^13^C NMR (100 MHz, DMSO-d6) δ (ppm): 32.3 (C-4 piperidinyl), 37.2 (C-3 & C-5 piperidinyl), 42.8 (CH_2_ benzyl), 50.9 (C-2 & C-6 piperidinyl), 71.8 (CH_2_ methyl), 126.1 (C-4 benzyl), 126.1 (C-4 pyridazinone), 128.5 (C-2 & C-6 benzyl), 129.3 (C-3 & C-5 benzyl), 129.3 (C-3 & C-5 phenyl), 129.7 (C-2 & C-6 phenyl), 130.5 (C-4 phenyl), 131.1 (C-1 phenyl), 134.8 (C-1 benzyl), 140.6 (C-5 pyridazinone), 142.9 (C-6 pyridazinone), 160.3 (C-3 pyridazinone). Calcd for C_23_H_25_N_3_O (359.46): C, 76.85; H, 7.01; N, 11.69; Found: C, 77.04; H, 7.22; N, 11.85%.

##### **2-(Morpholinomethyl)-6-(4-tolyl) pyridazin-3(2*H*)-one** (**2c**)

Yield: 76%, m.p. 168–170 °C; IR (KBr), cm^-1^: 3039 (arom.CH), 2831 (aliph.CH), 1651 (C = O);^1^H NMR (400 MHz, DMSO-d_6_) δ (ppm): 2.34 (s, 3 H, methyl CH_3_), 2.65 (m, 4 H, 2 CH_2_, C3 & C5 of morpholine), 3.54 (m, 4 H, 2 CH_2_, C2 and C6 of morpholine), 4.98 (s, 2h, methylene CH_2_), 7.04 (d, *J* = 12hz, 1 H, ArH of C5 pyridazinone), 7.28 (d, *J* = 8 Hz, 2h, ArH), 7.76 (d, *J* = 8 Hz, 2h, ArH), 7.99 (d, *J* = 12hz, 1 H, ArH of C4 pyridazinone);^13^C NMR (100 MHz, DMSO-d6) δ (ppm): 21.2 (CH_3_ tolyl ), 50.8 (C-3 & C-5 morpholinyl), 66.6 (CH_2_ methylene), 71.5 (C-2 & C-6 morpholinyl), 126.1 (C-4 pyridazinone), 129.9 (C-2 & C-6 tolyl), 130.5 (C-3 & C-5 tolyl), 131.2 (C-1 tolyl), 132.0 (C-4 tolyl), 139.4 (C-5 pyridazinone), 143.2 (C-6 pyridazinone), 160.3 (C = O pyridazinone). Calcd for C_16_H_19_N_3_O_2_ (285.34): C, 67.35; H, 6.71; N, 14.73; Found: C, 67.52; H, 6.83; N, 14.97%.

##### **2-((4-Benzylpiperidin-1-yl) methyl)-6-(4-tolyl) pyridazin-3(2*H*)-one** (**2d**)

Yield: 65%, m.p. 152–154 °C; IR (KBr), cm^-1^: 3024 (arom.CH), 2843 (aliph.CH), 1651 (C = O);^1^H NMR (400 MHz, DMSO-d_6_) δ (ppm): 1.09–1.17 (m, 2h, CH_2_, C3 of piperidine), 1.32–1.33 (m, 1 H, CH, C4 of piperidine), 1.47 (d, *J* = 12hz, 2h, CH_2_, C2 of piperidine), 2.21 (t, *J* = 8 Hz, 2h, CH_2_, C6 of piperidine), 2.32 (s, 3 H, methyl CH_3_), 2.42 (d, *J* = 4 Hz, 2h, CH_2_, C5 of piperidine), 3.0 (d, *J* = 12hz, 2h, benzyl CH_2_), 3.35 (s, 2h, methylene CH_2_), 7.01 (d, *J* = 12hz, 1 H, ArH of C5 pyridazinone), 7.07–7.14 (m, 3 H, ArH), 7.22 (t, *J* = 8 Hz, 2h, ArH), 7.27 (d, *J* = 8 Hz, 2h, ArH), 7.74 (d, *J* = 8 Hz, 2h, ArH), 7.98 (d, *J* = 8 Hz, 1 H, ArH of C4 of pyridazinone);^13^C NMR (100 MHz, DMSO-d6) δ (ppm): 21.2 (CH_3_ tolyl), 32.2 (C-4 piperidinyl), 37.2 (C-3 & C-5 piperidinyl), 42.8 (CH_2_ benzyl), 50.9 (C-2 & C-6 piperidinyl), 71.8 (CH_2_ methylene), 126.07 (C-4 benzyl), 126.08 (C-4 pyridazinone), 128.5 (C-2 & C-6 benzyl), 129.3 (C-3 & C-5 benzyl), 129.9 (C-2 & C-6 tolyl), 130.5 (C-3 & C-5 tolyl), 131.0 (C-1 tolyl), 132.1 (C-1 benzyl), 139.3 (C-4 tolyl), 140.6 (C-5 pyridazinone), 142.9 (C-6 pyridazinone), 160.2 (C-3 pyridazinone). Calcd for C_24_H_27_N_3_O (373.49): C, 77.18; H, 7.29; N, 11.25; Found: C, 77.40; H, 7.63; N, 11.52%.

##### **6-(4-Bromophenyl)-2-(morpholinomethyl) pyridazin-3(2*H*)-one** (**2e**)

Yield: 87%, m.p. 176–178 °C; IR (KBr), cm^-1^: 3051 (arom.CH), 2850 (aliph.CH), 1654 (C = O), 501 (C-Br);^1^H NMR (400 MHz, DMSO-d_6_) δ (ppm): 2.64 (t, *J* = 8 Hz, 4 H, C3 & C5 of morpholine), 3.54 (t, *J* = 8 Hz, 4 H, C2 & C6 morpholine), 4.98 (s, 2h, methylene CH_2_), 7.07 (d, *J* = 12hz, 1 H, ArH of C5 pyridazinone), 7.67 (d, *J* = 12hz, 2h, ArH), 7.81 (d, *J* = 8 Hz, 2h, ArH), 8.03 (d, *J* = 8 Hz, 1 H, ArH of C4 pyridazinone);^13^C NMR (100 MHz, DMSO-d6) δ (ppm): 50.7 (C-3 & C-5 morpholinyl), 66.6 (CH_2_ methylene), 71.6 (C-2 & C-6 morpholinyl), 123.4 (C-4 phenyl), 128.3 (C-4 pyridazinone), 131.1 (C-2 & C-6 phenyl), 131.16 (C-3 & C-5 phenyl), 132.3 (C-1 phenyl), 134.0 (C-5 pyridazinone), 142.2 (C-6 pyridazinone), 160.3 (C = O pyridazinone). Calcd for C_15_H_16_BrN_3_O_2_ (350.21): C, 51.44; H, 4.60; N, 12; Found: C, 51.72; H, 4.71; N, 12.13%.

##### **2-((4-Benzylpiperidin-1-yl) methyl)-6-(4-bromophenyl) pyridazin-3(2*H*)-one** (**2f**)

Yield: 58%, m.p. 125–127 °C; IR (KBr), cm^-1^: 3020 (arom.CH), 2846 (aliph.CH), 1651 (C = O), 590 (C-Br);^1^H NMR (400 MHz, DMSO-d_6_) δ (ppm): 1.08–1.17 (m, 2h, CH_2_, C3 of piperidine), 1.30–1.33 (broad m, 1 H, CH, C4 of piperidine), 1.47 (d, *J* = 12hz, 2h, CH_2_, C5 of piperidine), 2.20 (t, *J* = 12hz, 2h, CH_2_, C2 of piperidine), 2.41 (d, *J* = 8 Hz, 2h, CH_2_, C6 of piperidine), 2.99 (d, *J* = 12hz, 2h, benzyl CH_2_), 4.97 (s, 2h, methylene CH_2_), 7.03 (d, *J* = 12hz, 1 H, ArH of C5 of pyridazinone), 7.06 (d, *J* = 4 Hz, 2h, ArH), 7.12 (t, *J* = 16 Hz, 1 H, ArH), 7.21 (t, *J* = 16 Hz, 2h, ArH), 7.65 (d, *J* = 8 Hz, 2h, ArH), 7.79 (d, *J* = 8 Hz, 2h, ArH), 8.01 (d, *J* = 8 Hz, 1 H, ArH of C4 pyridazinone);^13^C NMR (100 MHz, DMSO-d6) δ (ppm): 32.2 (C-4 piperidinyl), 37.2 (C-3 & C-5 piperidinyl), 42.8 (CH_2_ benzyl), 50.8 (C-2 & C-6 piperidinyl), 71.9 (CH_2_ methylene), 123.3 (C-4 phenyl), 126.1 (C-4 pyridazinone), 128.1 (C-2 & C-6 benzyl), 128.5 (C-3 & C-5 benzyl), 129.34 (C-2 & C-6 phenyl), 130.6 (C-3 & C-5 phenyl), 130.8 (C-1 phenyl), 132.3 (C-1 benzyl), 134.1 (C-4 phenyl), 140.6 (C-5 pyridazinone), 141.9 (C-6 pyridazinone), 160.2 (C-3 pyridazinone). Calcd for C_23_H_24_BrN_3_O (438.36): C, 63.02; H, 5.52; N, 9.59; Found: C, 63.21; H, 5.70; N, 9.76%.

##### **6-(4-Hydroxyphenyl)-2-(morpholinomethyl) pyridazin-3(2*H*)-one** (**2g**)

Yield: 60%, m.p. 260–262 ^0^C; IR (KBr), cm^-1^: 3653 (OH), 3070 (arom.CH), 2827 (aliph.CH), 1651 (C = O);^1^H NMR (400 MHz, DMSO-d_6_) δ (ppm): 2.39–2.72 (m, 4 H, 2CH_2,_ overlapped with DMSO, C3 & C5 of piperidine), 3.43–3.63 (broad m, 4 H, 2CH_2_, C2 & C6 of piperidine), 4.96 (s, 2h, methylene CH_2_), 6.84–6.94 (m, 2h, ArH), 7.00 (d, *J* = 12hz, 1 H, ArH of C5 pyridazinone), 7.67–7.72 (m, 2h, ArH), 7.94 (t, *J* = 16 Hz, 1 H, ArH of C4 pyridazinone), 9.84 (s, 1 H, OH, D_2_O exchangeable);^13^C NMR (100 MHz, DMSO-d6) δ (ppm): 50.7 (C-2 & C-6 morpholinyl), 66.6 (C-3 & C-5 morpholinyl), 71.4 (CH_2_ methylene), 116.1 (C-3 & C-5 phenol), 125.9 (C-1 phenol), 127.8 (C-4 pyridazinone), 130.4 (C-2 & C-6 phenol), 143.5 (C-5 pyridazinone), 144.6 (C-6 pyridazinone), 159.1 (C-3 pyridazinone), 160.3 (C-4 phenol). Calcd for C_15_H_17_N_3_O_3_ (287.31): C, 62.71; H, 5.96; N, 14.63; Found: C, 62.53; H, 6.14; N, 14.85%; HR-MS (ESI): calcd for C_15_H_17_N_3_O_3_ (287.12); found (287.12).

##### **2-((4-Benzylpiperidin-1-yl) methyl)-6-(4-hydroxyphenyl) pyridazin-3(2*H*)-one** (**2h**)

Yield: 48%, m.p. 167–169 °C; IR (KBr), cm^-1^: 3024 (arom.CH), 2846 (aliph.CH), 1654 (C = O);^1^H NMR (400 MHz, DMSO-d_6_) δ (ppm): 1.08–1.15 (m, 2h, CH_2_, C3 of piperidine), 1.35 (broad s,1 H, CH, C4 of piperidine), 1.47–1.50 (m, 2h, CH_2_, C5 of piperidine), 2.19 (t, *J* = 12hz, 2h, CH_2_, C2 of piperidine), 2.43 (t, *J* = 16 Hz, 2h, CH_2_, overlapped with DMSO, C6 of piperidine), 3.00 (d, *J* = 12hz, 2h, benzyl CH_2_,), 4.94 (s, 2h, methylene CH_2_), 6.85 (d, *J* = 8 Hz, 2h, ArH), 6.95 (d, *J* = 12hz, 1 H, ArH of C5 pyridazinone), 7.08 − 7.15 (m, 3 H, ArH), 7.23 (t, *J* = 8 Hz, 2h, ArH), 7.69 (d, *J* = 8 Hz, 2h, ArH), 7.92 (d, *J* = 8 Hz, 1 H, ArH of C4 pyridazinone), 9.74 (s, 1 H, OH, D_2_O exchangeable);^13^C NMR (100 MHz, DMSO-d6) δ (ppm): 32.2 (C-4 piperidinyl), 37.2 (C-3 & C-5 piperidinyl), 42.7 (CH_2_ benzyl), 50.9 (C-2 & C-6 piperidinyl), 71.6 (CH_2_ methylene), 116.1 (C-3 & C-5 phenol), 126.1 (C-1 phenol), 127.6 (C-4 benzyl), 128.6 (C-4 pyridazinone), 129.3 (C-2 & C-6 benzyl), 130.4 (C-3 & C-5 benzyl), 130.9 (C-2 & C-6 phenol), 140.6 (C-1 benzyl), 143.2 (C-5 pyridazinone), 159.0 (C-6 pyridazinone), 159.1 (C-3 pyridazinone), 160.2 (C-4 phenol). Calcd for C_23_H_25_N_3_O_2_ (375.46): C, 73.57; H, 6.71; N, 11.19; Found: C, 73.40; H, 6.89; N, 11.45%.

##### **2-(Morpholinomethyl)-6-(4-nitrophenyl) pyridazin-3(2*H*)-one** (**2i**)

Yield: 90%, m.p. 182–184 °C; IR (KBr), cm^-1^: 3051 (arom.CH), 2835 (aliph.CH), 1681 (C = O);^1^H NMR (400 MHz, DMSO-d_6_) δ (ppm): 2.65–2.82 (m, 4 H, C3 & C5 of morpholine), 3.54 (m, 4 H, C2 & C6 of morpholine), 5.02 (s, 2h, methylene CH_2_), 7.12 (d, *J* = 8 Hz, 1 H, ArH of C5 pyridazinone), 8.10 (d, *J* = 8 Hz, 1 H, ArH of C4 pyridazinone), 8.15 (d, *J* = 8 Hz, 2h, ArH), 8.30 (d, *J* = 8 Hz, 2h, ArH);^13^C NMR (100 MHz, DMSO-d6) δ (ppm): 50.7 (C-3 & C-5 morpholinyl), 66.6 (CH_2_ methylene), 71. 8 (C-2 & C-6 morpholinyl), 124.5 (C-4 pyridazinone), 127.3 (C-2 & C-6 nitrophenyl), 130.7 (C-3 & C-5 nitrophenyl), 131.3 (C-1 nitrophenyl), 140.8 (C-4 nitrophenyl), 141.3 (C-5 pyridazinone), 148.1 (C-6 pyridazinone), 160.3 (C = O pyridazinone). Calcd for C_15_H_16_N_4_O_4_ (316.31): C, 56.96; H, 5.10; N, 17.71; Found: C, 60.12; H, 5.17; N, 17.94%. HR-MS (ESI): calcd for C_15_H_16_N_4_O_4_ (316.11); found (316.01).

##### **2-((4-Benzylpiperidin-1-yl) methyl)-6-(4-nitrophenyl) pyridazin-3(2*H*)-one** (**2j**)

Yield: 70%, m.p. 143–145 °C; IR (KBr), cm^-1^: 3055 (arom.CH), 2912 (aliph.CH), 1662 (C = O);^1^H NMR (400 MHz, DMSO-d_6_) δ (ppm): 1.11–1.23 (m, 2h, CH_2_, C3 of piperidine), 1.36–1.38 (broad m, 1 H, CH, C4 of piperidine), 1.52 (d, *J* = 12hz, 2h, CH_2_, C5 of piperidine), 2.25 (t, *J* = 24 Hz, 2h, CH_2_, C2 of piperidine), 2.45–2.46 (d, *J* = 4 Hz, 2h, CH_2_, C6 of piperidine), 3.05 (d, *J* = 12hz, 2h, benzyl CH_2_), 5.04 (s, 2h, methylene CH_2_), 7.08–7.12 (m, 3 H, ArH), 7.15 (d, *J* = 4 Hz, 1 H, ArH of C5 pyridazinone), 7.24 (t, *J* = 16 Hz, 2h, ArH), 8.13 (d, *J* = 4 Hz, 1 H, ArH of C4 pyridazinone), 8.16 (d, *J* = 8 Hz, 2h, ArH), 8.33 (d, *J* = 8 Hz, 2h, ArH);^13^C NMR (100 MHz, DMSO-d6) δ (ppm): 32.2 (C-4 piperidinyl), 37.2 (C-3,5 piperidinyl), 42.7 (CH_2_ benzyl), 50.8 (C-2 & C-6 piperidinyl), 72.1 (CH_2_ methylene), 124.5 (C-3 & C-5 nitrophenyl), 126.1 (C-4 benzyl), 127.3 (C-4 pyridazinone), 128.5 (C-2 & C-6 benzyl), 129.3 (C-3 & C-5 benzyl), 130.6 (C-2 & C-6 nitrophenyl), 131.1 (C-1 benzyl), 131.8 (C-1 nitrophenyl), 140.6 (C-5 pyridazinone), 148.1 (C-4 nitrophenyl), 160.3 (C-6 pyridazinone), 160.7 (C-3 pyridazinone). Calcd for C_23_H_24_N_4_O_3_ (404.46): C, 68.30; H, 5.98; N, 13.85; Found: C, 68.53; H, 6.12; N, 14.03%.

### Biological evaluation

Biological evaluation was done in the Medical Pharmacology department, Kasralainy Faculty of medicine, Cairo University. Fifty, Healthy, mature, adult, male Wistar Albino rats each weighing 180–200 g were used in the present study. Animals were obtained from Kasralainy animal house (Giza, Egypt). They were reared in a closed system and were kept under standard housing conditions: humidity (60 ± 10%), room temperature (25 ± 2 ^0^C) and a light/dark cycle of 12/12h. Animals were allowed a free access to food and water provided. Adequate measures were taken to minimize pain or discomfort of animals and all procedures were performed in accordance with the direction of “Research Ethical Committee”, Faculty of Pharmacy, Cairo University which comply with ARRIVE guidelines for use and care of laboratory animals with approval number REC-FPCU-3529.

#### Vasorelaxant activity of the thoracic aortic rings with intact endothelium

All target compounds were screened for their vasorelaxant activity on thoracic aorta according to the reported procedure^10,19,20,27–29^. The tested compounds were dissolved in dimethylsulfoxide (DMSO) as stock solution (10 mL of 0.005 M). Rats were euthanized using 150 mg intraperitoneal injection of phenobarbitone followed by cervical dislocation. Thoracic aorta were dissected out from rats into a petri dish filled with cold Krebs–Henseleit buffer of the following composition (mM): NaCl, 115; KC1, 4.7; CaCl_2_, 2.5; MgC1_2_, 1.2; NaHCO_3_, 25; KH_2_PO_4_, 1.2; and dextrose, 11.1, bubbled with carbogen gas of 95% O_2_ and 5% CO_2_ and adjusted to pH 7.4.

Briefly, vascular tissues were immediately freed from perivascular adipose tissue then cut into 3–4 mm long rings. Each aortic ring was mounted between two stainless steel triangular hooks and vertically suspended in 10 ml-organ baths **(Manufacturer: Panlab**,** Spain Model number: LE11200 (including a thermo regulated water pump)** containing Krebs–Henseleit buffer, where each ring was attached to a routinely calibrated isometric force displacement transducers (**Manufacturer: Panlab**,** Spain Model number: TRI201)** from one side, and to a stationary hook on the other side. All transducers were connected to a Quad Bridge Amp that is connected to a computer through a data acquisition system - PowerLab Data Acquisition: **Manufacturer: ADinstruments**,** Australia Model number: PowerLab 4/30 with LabChart Pro (Product# ML866/P)** were used to monitor the changes in tension (gm). The temperature in organ baths was maintained at 37 ^◦^C by a circulating water bath system. Aortic rings were kept under a constant tension of 1gm for 45 min. After this equilibration time, a single concentration of 80 mM KCl was used to check the viability of the isolated aortic rings. The contractile tone of each aortic ring was assessed initially by phenylephrine 2 µg of 1/100.000 subsequently with Ach (10 µg of 1/100.000 to evaluate the integrity of the endothelium before further experiments exposure to starting dose 2 µg of 1/100.000 freshly prepared phenylephrine solution. The duration of contraction was 2 min and time interval between doses was 1 min then repeating with 4 µg, 8 µg and16µg of phenylephrine constructing a dose response curve. A submaximal concentration of the standard contractile agent PE will be added and allowed to produce a stable contractile response (plateau). Then, relaxation responses of cumulative concentrations of the tested compounds were recorded in PE-pre-contracted tissues.

Additionally, nitroglycerin was selected as a positive control drug and the responses to nitroglycerin (conc to conc M), an endothelium-dependent nitro- dilator, were generated in a separate set or aortic rings pre-contracted with PE Each concentration was allowed a sufficient time to reach a stable response (plateau) before adding the next dose. The relaxant response of each concentration was calculated as percent inhibition of PE-induced contraction according to the following equation: % relaxation = Decrease in tension after adding a certain concentration / Tension developed by phenylephrine before adding any drugs × 100. Moreover, the vasorelaxant effects was seem to be reversible because a washout period of 30 min allowed a progressive and complete recovery of both contractility of the rings and vasorelaxation.

Tested compounds were dissolved in DMSO to prepare serial dilutions (0.001–10 mM). Equivalent amounts of DMSO were added to aortic rings in a separate experiment to act as vehicle controls which did not affect the contractile response of aorta. Equivalent amounts of Data were deducted from experimental files using built-in functions of the Lab Chart^®^ Pro 7 software for Windows^®^ and the relaxation responses were calculated in accordance with the previous equation. Data were manifested as mean ± standard error of the mean of 5 independent experiments. Nonlinear regression was applied to the concentration– effect curves using a built-in three parameter dose–response curve equation supplied with Graph Pad Prism^®^ 7 Software for Windows.

### Molecular modeling study

#### 3D QSAR pharmacophore study

All compounds were build using Accelry’s Discovery Studio 4.0 window and optimized using CHARMm force field. The compounds were divided randomly into training and test set were the training set constitutes 80% of the total compounds number. The training set molecules were submitted to the HypoGen algorithm of DS 4.0. among the 11 features available for selection, the features H-bond acceptor, H-bond donor, ring aromatic, positive ionizable and hydrophobic features present in the training set were selected for pharmacophore generation. 3D QSAR generation protocol was applied in Discovery Studio software. The outcome of HypoGen results in a large number of hypotheses, the cost analysis was applied to evaluate the hypotheses. Evaluation of the hypothesis was performed on the basis of three cost values, null, fixed and total costs. The fixed cost represents the simplest model that fits all data perfectly while the null cost represents a model with no features, whereas the total cost is the summation of the weight, error and configuration costs. RMS (root mean square difference) between the estimated and predicted activity of the training set increases the error cost, the feature weights in the model as it deviates from the ideal value of 2.0 increase the weight cost while the configuration cost depends on the hypothetical space complexity to be analyzed. The hypothesis statistical significance increase as the total cost approach the fixed cost value and the cost difference between null and fixed cost is high^30^.

#### ADME and toxicity studies

All the synthesized compounds were subjected to in silico ADMET studies through utilizing Accelrys Discovery studio 4.5 ADMET descriptors algorithm. Different pharmacokinetic properties such as aqueous solubility, blood brain barrier penetration, CYP2D6 inhibition as well as human intestinal absorption were predicted^31–34^. Moreover extensive toxicity profile prediction of the synthesized compounds were implemented using the Discovery studio^35^.

#### Statistical analysis

Statistical analysis were performed using Graph pad prism Version 8.4.3 Software (GraphPad Software; San Diego, CA, USA). Each EC_50_ of the different investigated drugs was calculated via nonlinear regression model. Data were expressed as the mean (M) ± standard deviation. One-way Analysis of variance and post hoc Dunnet’s test was used to assess significant differences between the EC_50_ and maximum percentage of relaxation means. *p* < 0.05 was considered statistically significant.

#### Study limitation

We examined our investigated compounds only on endothelium intact thoracic aortic rings so we didn’t assess the independent endothelium mechanism by which these drugs might have .Therefore we are going to do an experimental design on the most 3 potent drugs (**2e**, **2h**, **2j**) to assess the effect on endothelial denution on the percentage of relaxation with further validation of molecular mechanism by investigating NO release, mRNA expression and western blot expression for eNOS.

## Electronic supplementary material

Below is the link to the electronic supplementary material.

Supplementary Material 1

## Data Availability

All data generated or analyzed during this study are included in this published article and its supplementary information files.
