# Supplementary material for: Synthesis of potent vasodilating agents: in silico and in vitro evaluation of 6-(4-substitutedphenyl)-3-pyridazinone derivatives as potential hydralazine analogues
Source: Sci Rep. 2024 Nov 27;14:29514. doi: 10.1038/s41598-024-79697-1 (PMC11603188; doi:10.1038/s41598-024-79697-1)
Supplement: Supplementary file 1 — Supplementary Material 1 [file 41598_2024_79697_MOESM1_ESM.docx]

**Synthesis of potent vasodilating agents: *in silico* and *in vitro* evaluation of 6-(4-substitutedphenyl)-3-pyridazinone derivatives as potential hydralazine analogues**

Marian W. Aziz^1,*^, Khaled O. Mohamed^2,3^ , Doaa B. Farag^1^, Amira Karam Khalifa^4,5^, and Zeinab Mahmoud^2,*^.

^1^Pharmaceutical chemistry department, Faculty of Pharmacy, Misr International University.

^2^Pharmaceutical organic chemistry department, Faculty of Pharmacy, Cairo University.

^3^Pharmaceutical chemistry department, Faculty of Pharmacy, Sinai University (Arish branch).

^4^Medical pharmacology department, Faculty of Medicine (Kasr alainy), Cairo University.

^5^Medical Pharmacology department, Faculty of Medicine, Nahda University.

**Table of Contents**

| ***Figure*** | ***Spectral data*** | ***Page*** | ***Figure*** | ***Spectral data*** | ***Page*** |
| --- | --- | --- | --- | --- | --- |
| **S1** | IR Chart of 2a | 3 | **S18** | ^13^C NMR of 2f | 20 |
| **S2** | ^1^H NMR of 2a | 4 | **S19** | IR Chart of 2g | 21 |
| **S3** | ^13^C NMR of 2a | 5 | **S20** | ^1^H NMR of 2g | 22 |
| **S4** | IR Chart of 2b | 6 | **S21** | ^13^C NMR of 2g | 23 |
| **S5** | ^1^H NMR of 2b | 7 | **S22** | D_2_O of 2g | 24 |
| **S6** | ^13^C NMR of 2b. | 8 | **S23** | HR-MS of 2g | 25 |
| **S7** | IR Chart of 2c. | 9 | **S24** | IR Chart of 2h | 26 |
| **S8** | ^1^H NMR of 2c | 10 | **S25** | ^1^H NMR of 2h | 27 |
| **S9** | ^13^C NMR of 2c | 11 | **S26** | ^13^C NMR of 2h | 28 |
| **S10** | IR Chart of 2d | 12 | **S27** | D_2_O of 2h | 29 |
| **S11** | ^1^H NMR of 2d | 13 | **S28** | IR Chart of 2i | 30 |
| **S12** | ^13^C NMR of 2d. | 14 | **S29** | ^1^H NMR of 2i | 31 |
| **S13** | IR Chart of 2e | 15 | **S30** | ^13^C NMR of 2i | 32 |
| **S14** | ^1^H NMR of 2e | 16 | **S31** | HR-MS of 2i | 33 |
| **S15** | ^13^C NMR of 2e | 17 | **S32** | IR Chart of 2j | 34 |
| **S16** | IR Chart of 2f. | 18 | **S33** | ^1^H NMR of 2j | 35 |
| **S17** | ^1^H NMR of 2f. | 19 | **S34** | ^13^C NMR of 2j | 36 |

***Spectral Data***


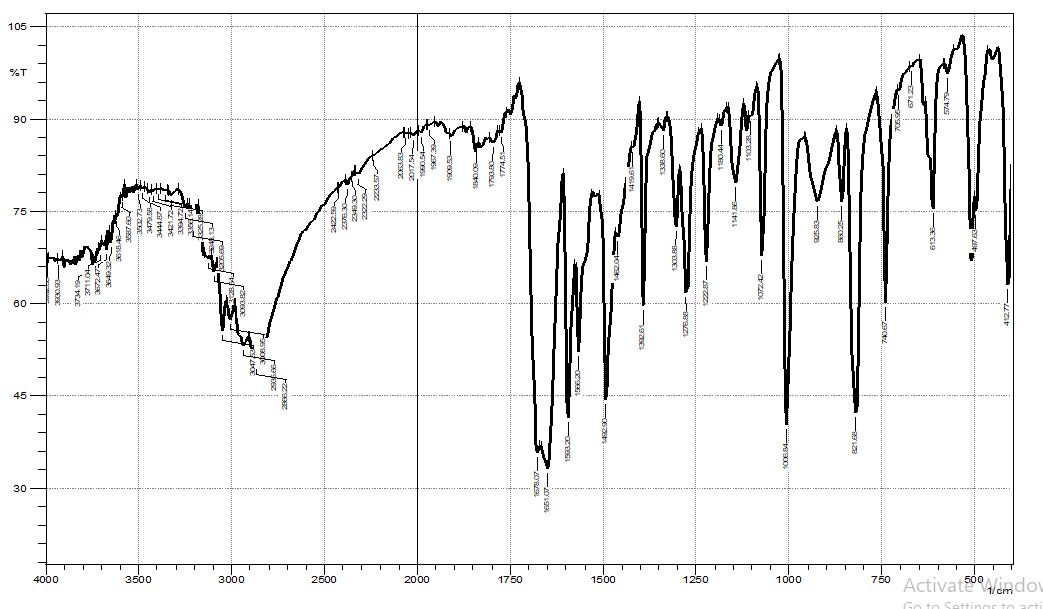


Figure S1. IR Chart of 2a.


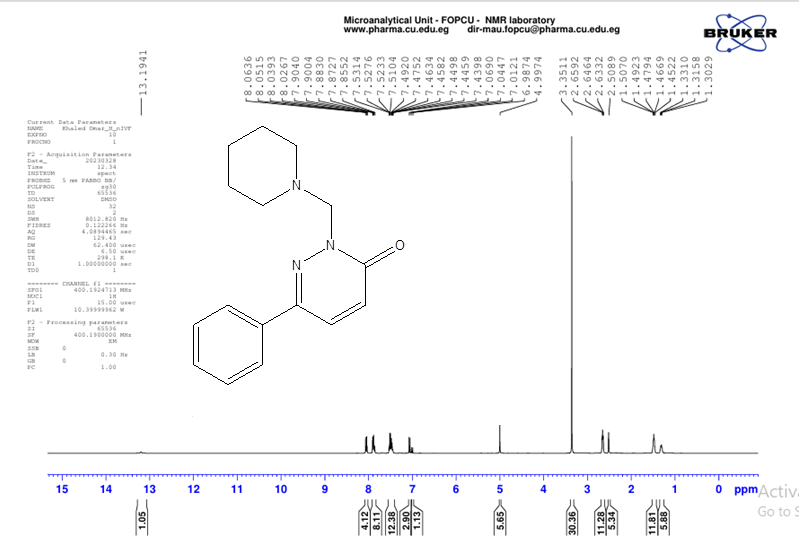


Figure S2. ^1^H NMR of 2a.


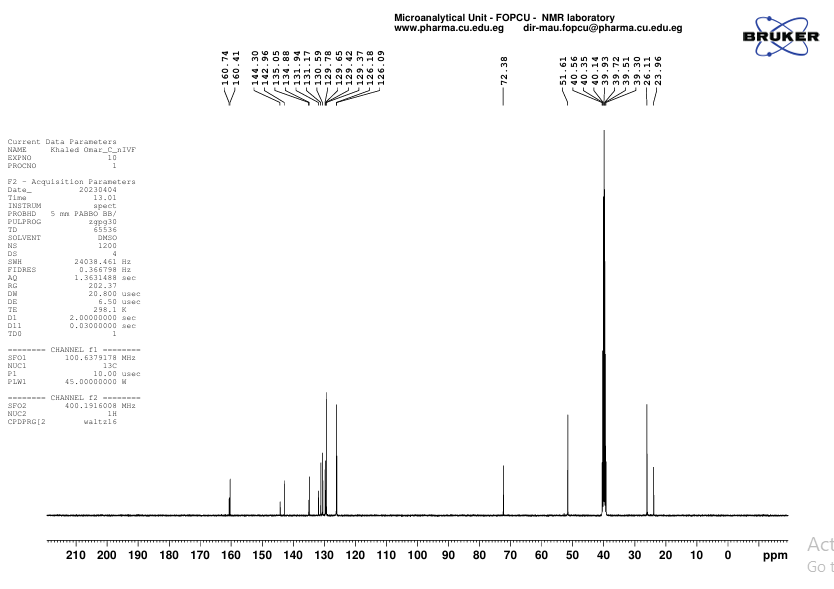


Figure S3. ^13^C NMR of 2a.


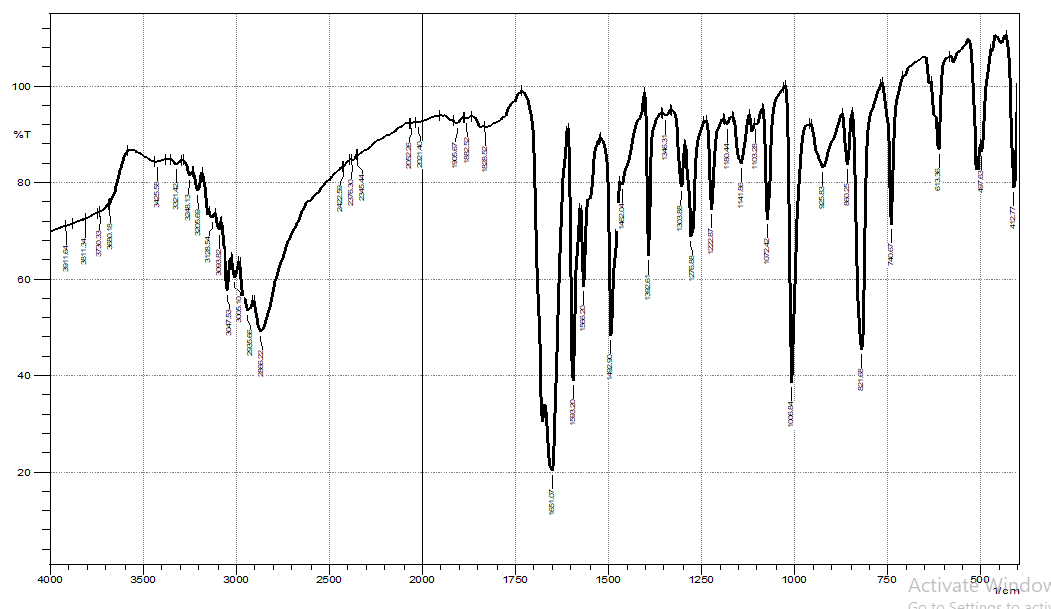
Figure S4. IR Chart of 2b.


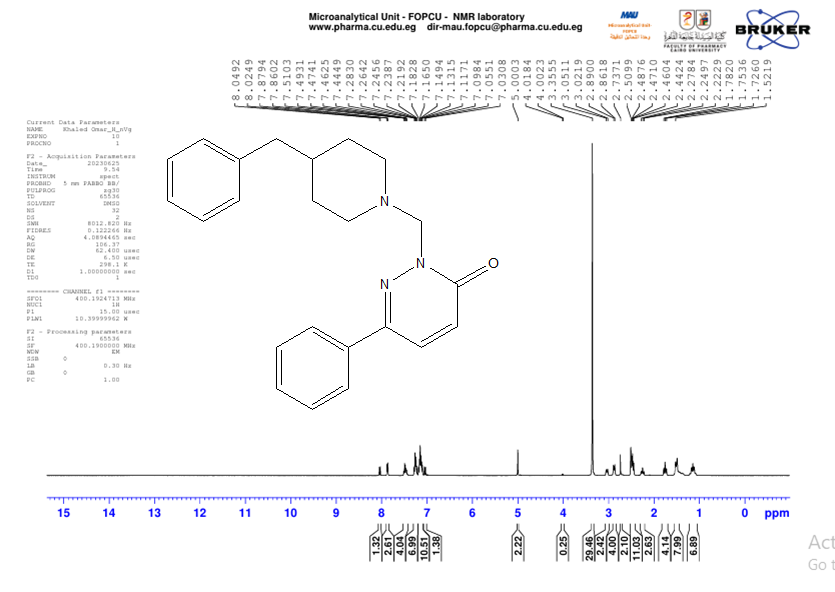
Figure S5. ^1^H NMR of 2b.


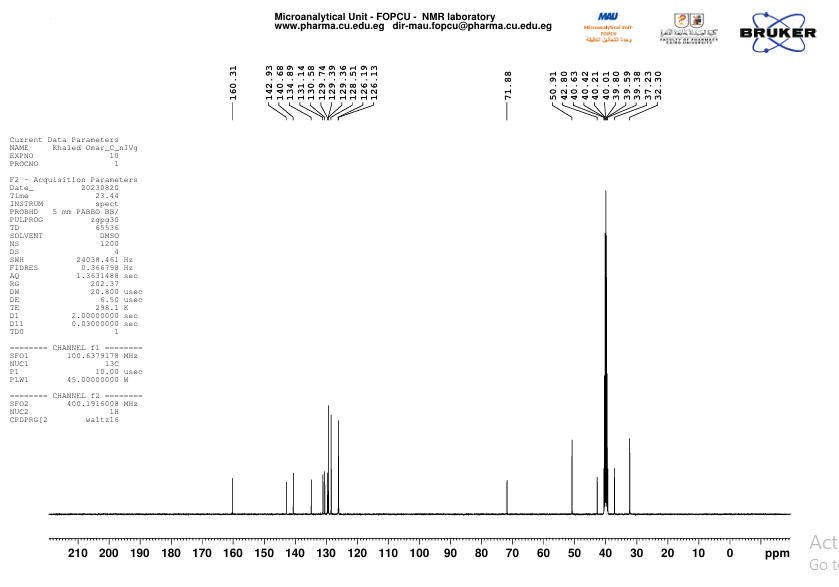
Figure S6. ^13^C NMR of 2b.


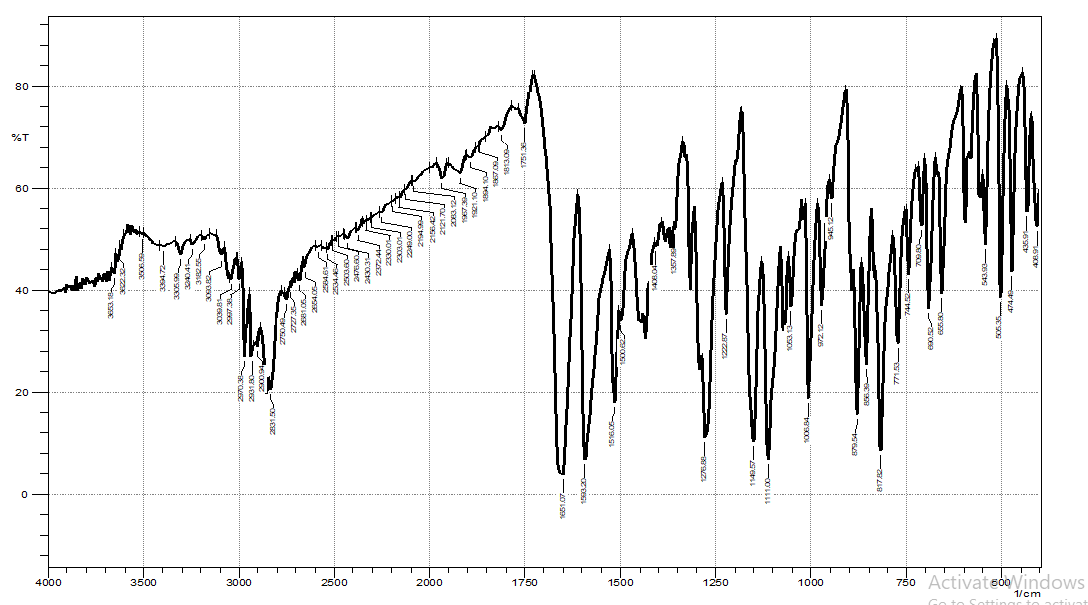
Figure S7. IR Chart of 2c.


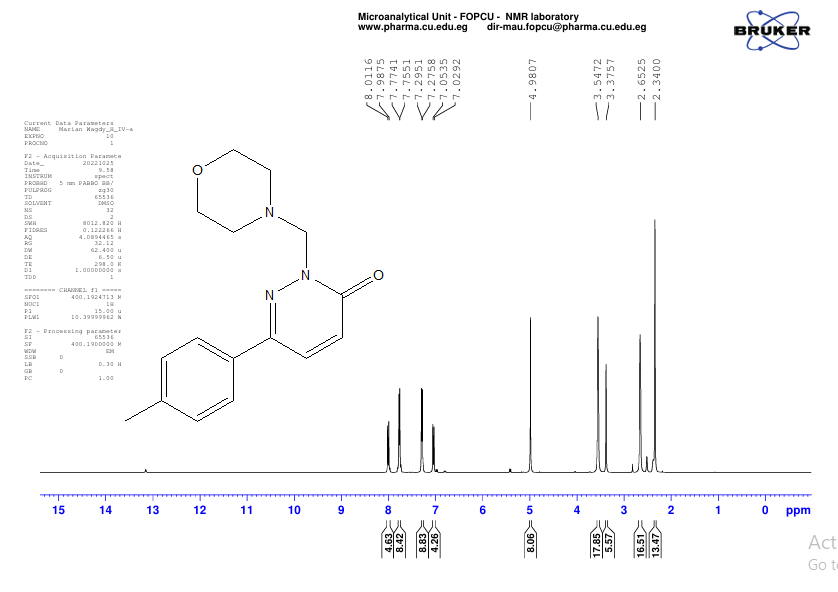
Figure S8. ^1^H NMR of 2c.


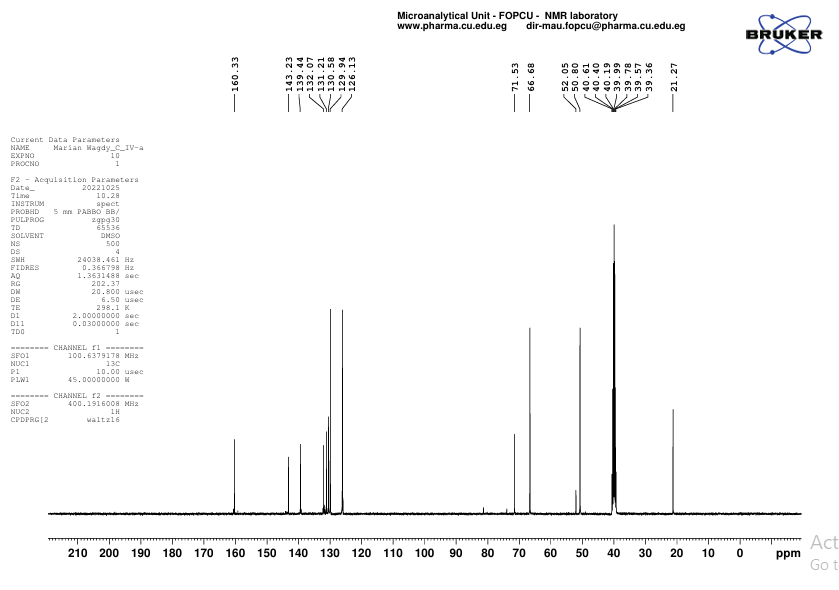
Figure S9. ^13^C NMR of 2c.


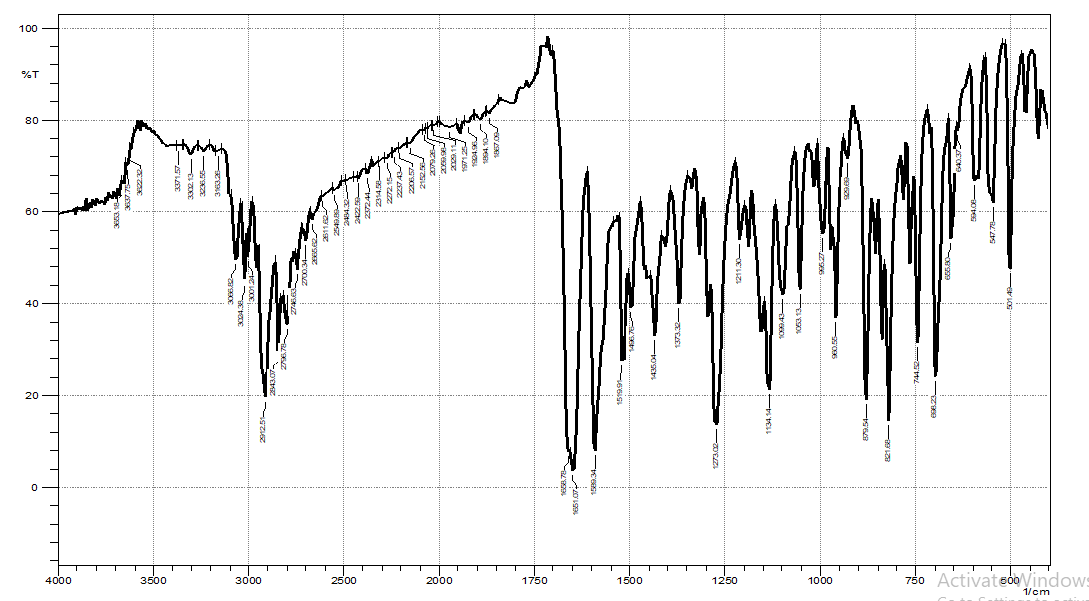
Figure S10. IR Chart of 2d.


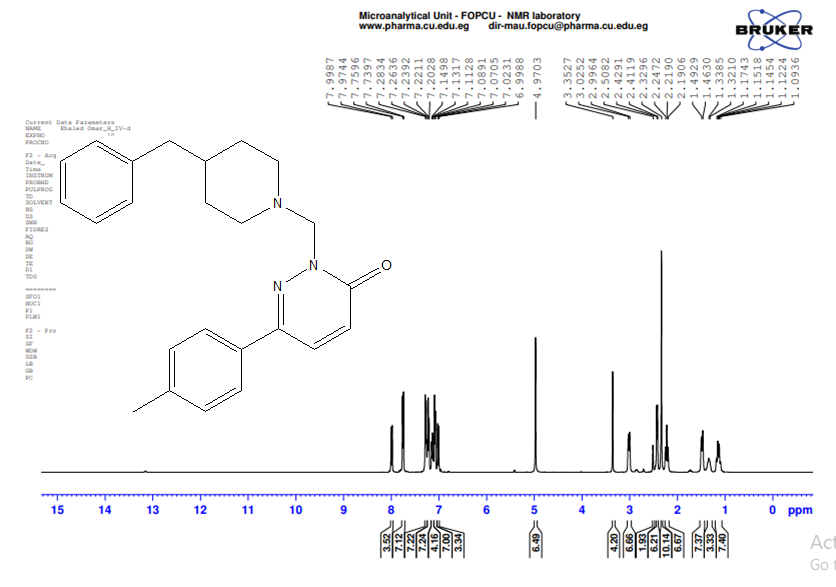
Figure S11. ^1^H NMR of 2d.


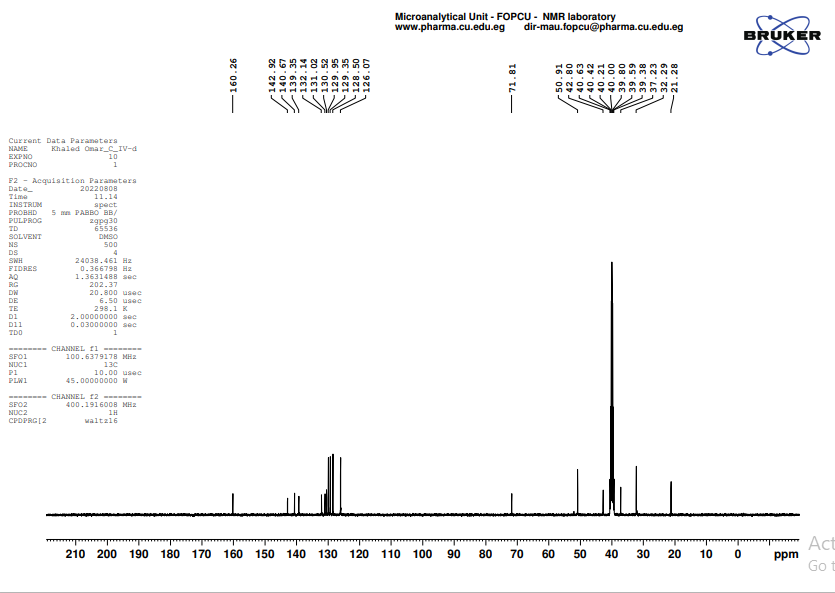


Figure S12. ^13^C NMR of 2d.


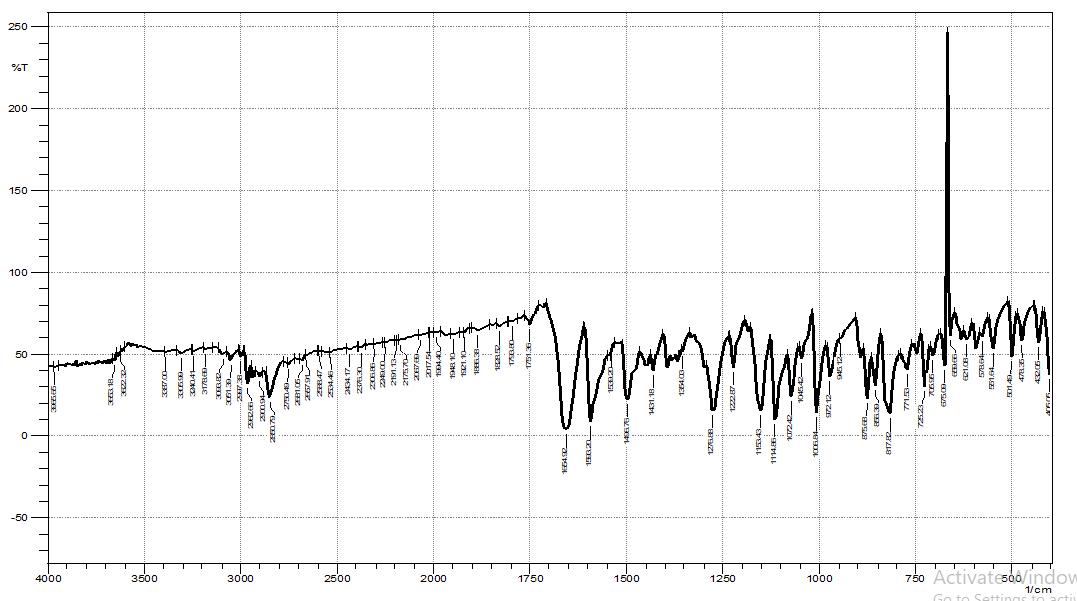
Figure S13. IR Chart of 2e.


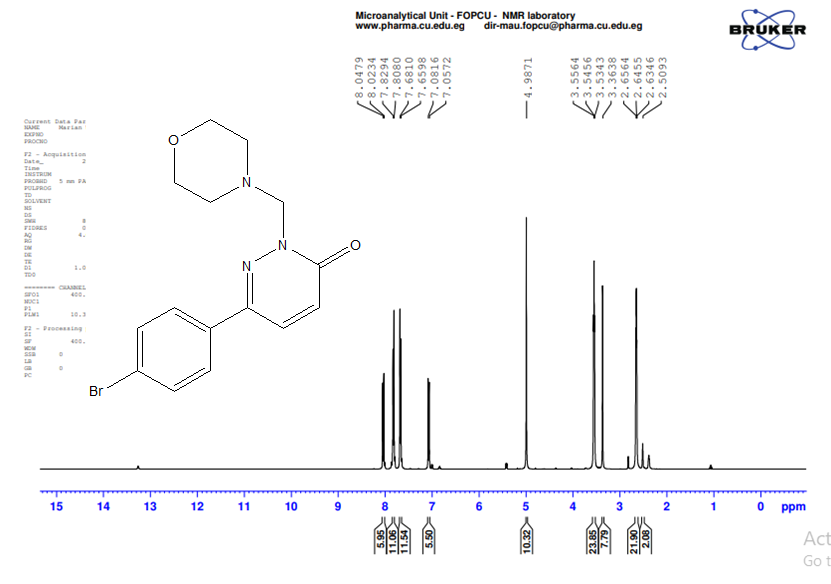
 Figure S14. ^1^H NMR of 2e.


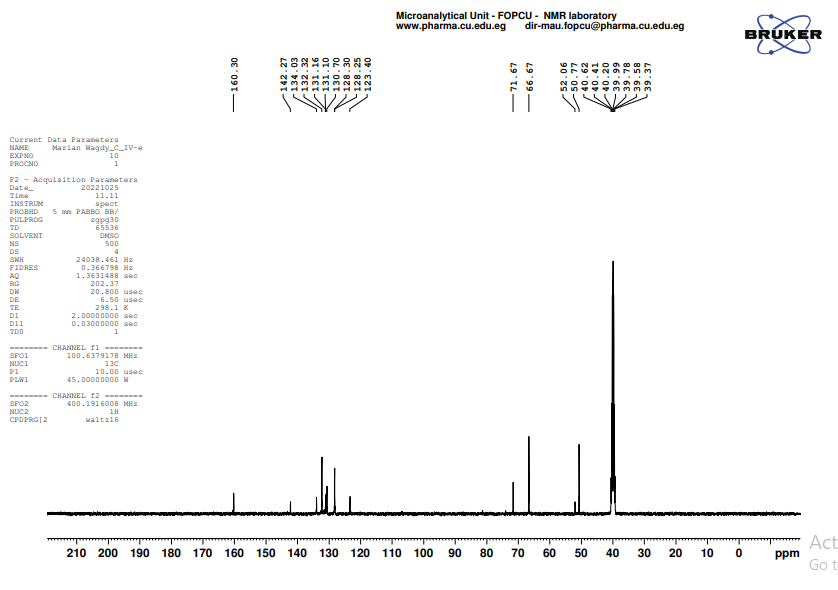
Figure S15. ^13^C NMR of 2e.


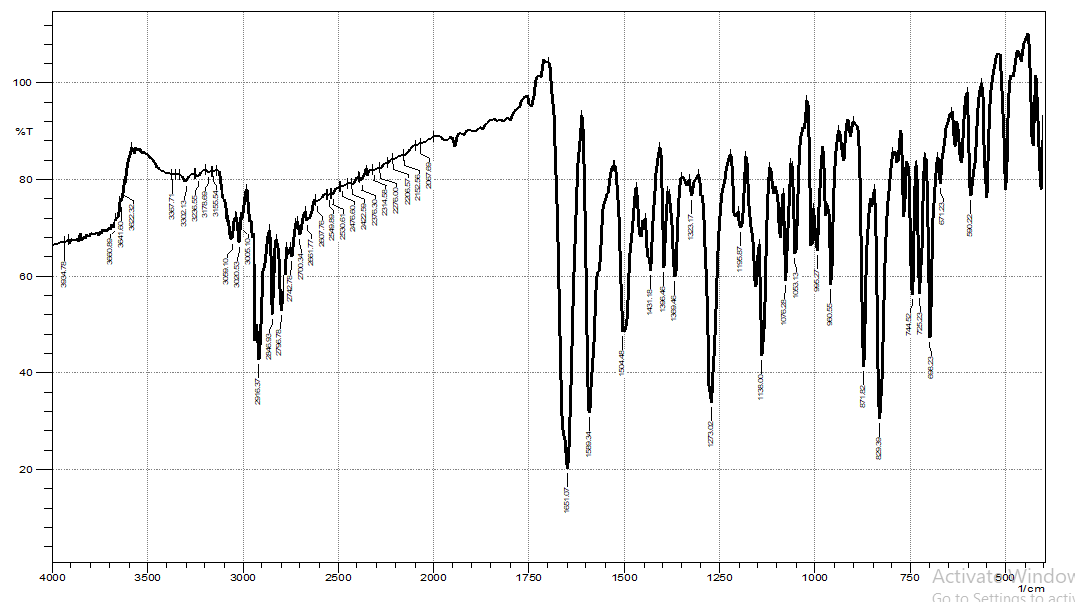
Figure S16. IR Chart of 2f.


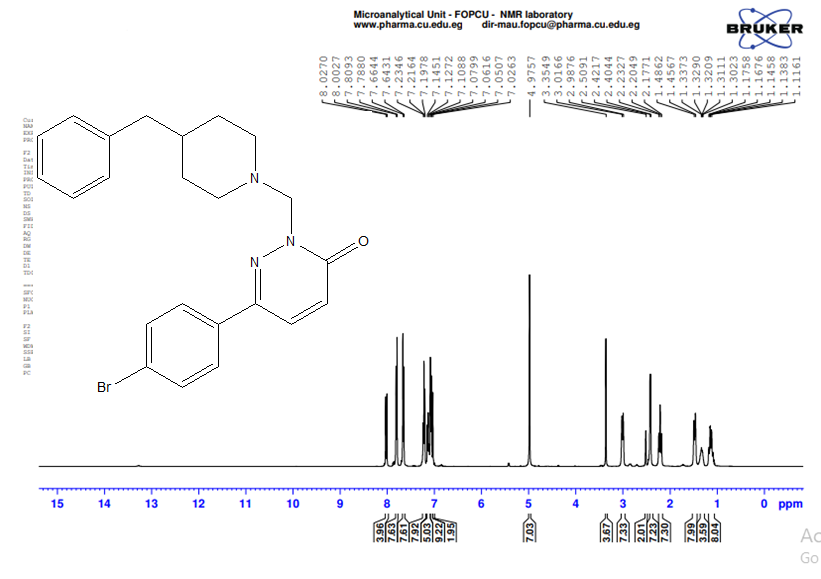
Figure S17. ^1^H NMR of 2f.


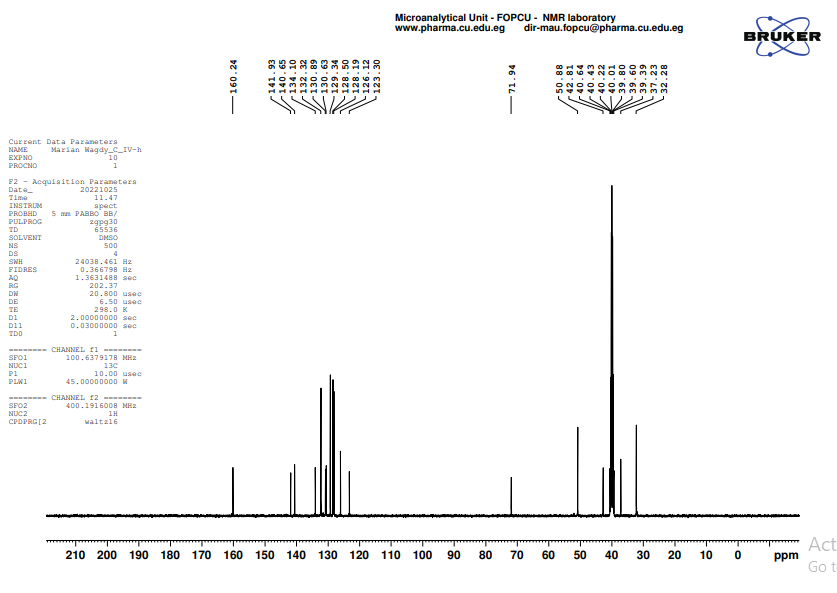
 Figure S18. ^13^C NMR of 2f.


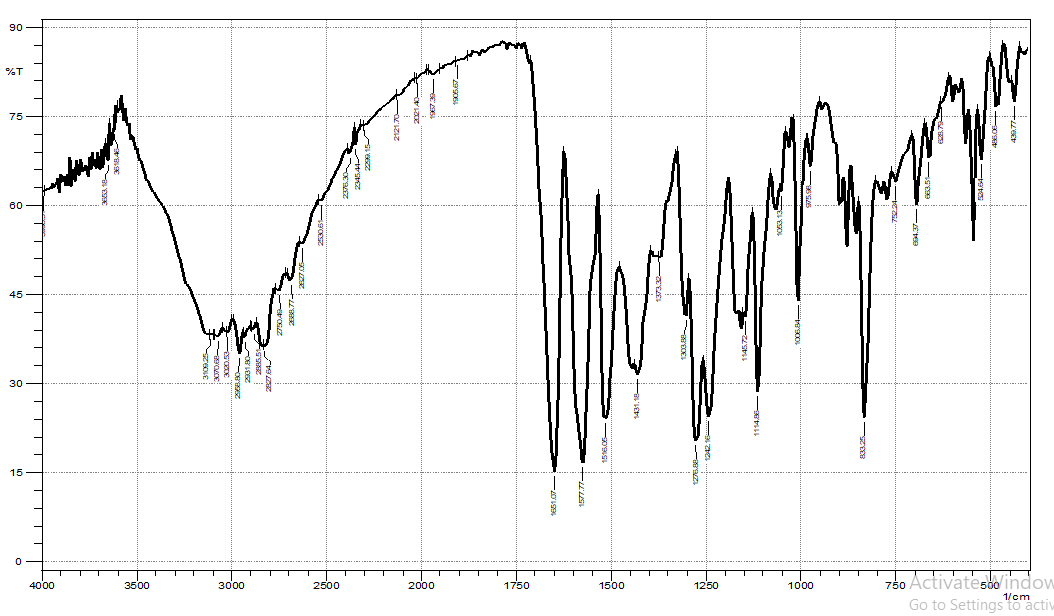
 Figure S19. IR Chart of 2g.


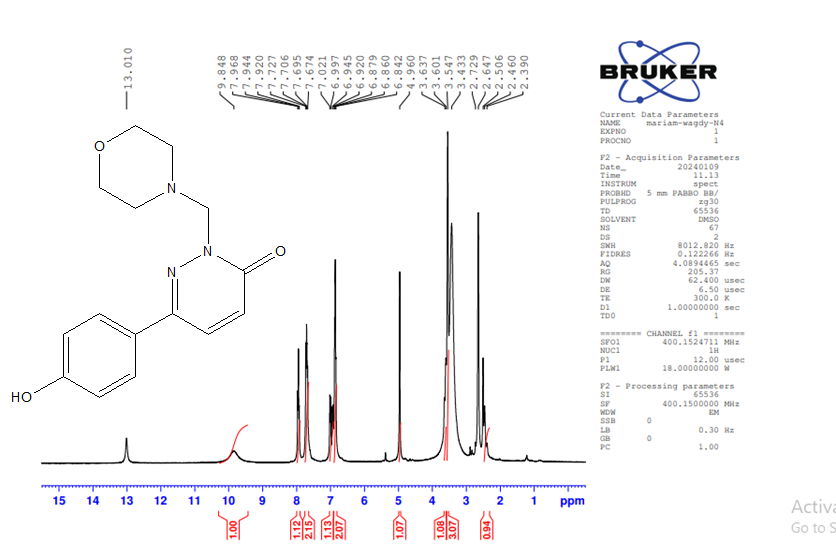
Figure S20. ^1^H NMR of 2g.


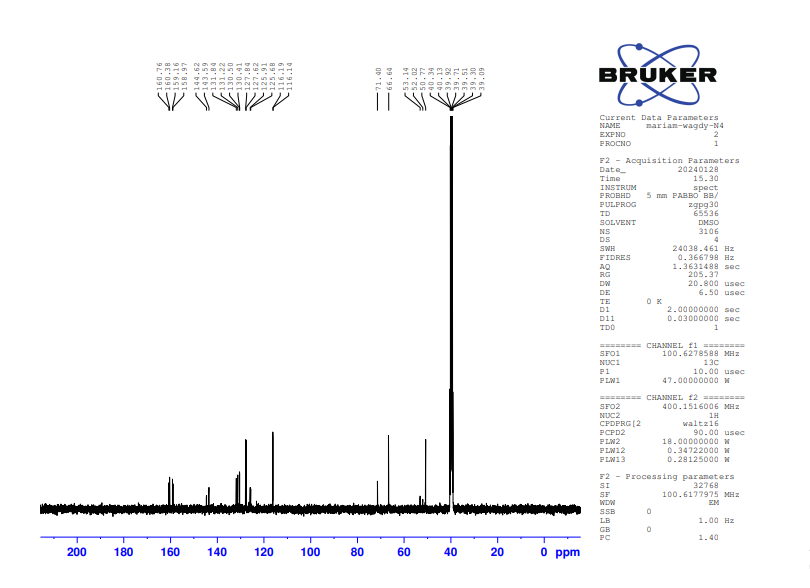
Figure S21. ^13^C NMR of 2g.


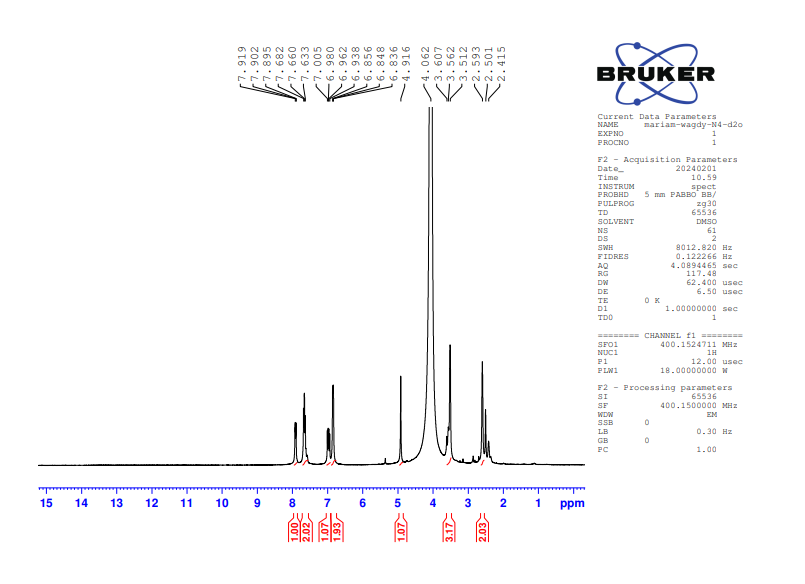
Figure S22. D_2_O of 2g.


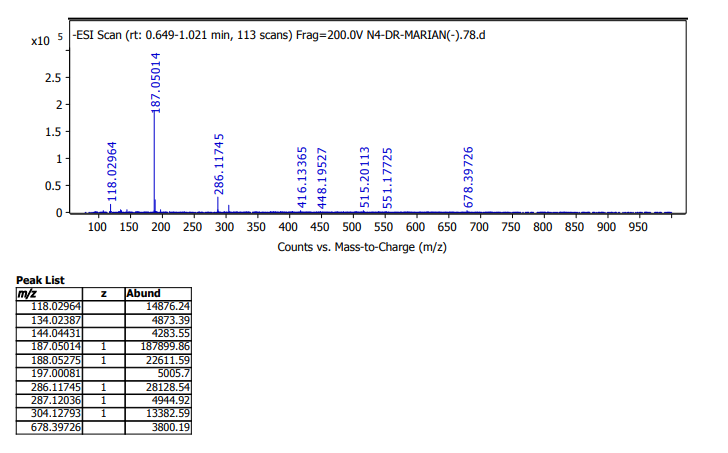


Figure S23. HR-MS Spectra of 2g.


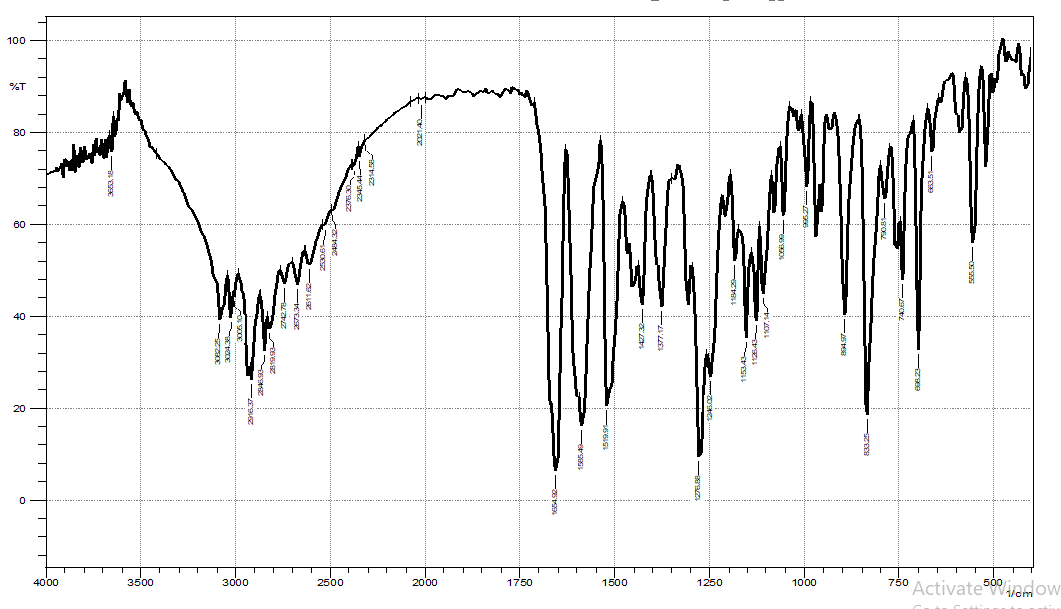
Figure S24. IR Chart of 2h.


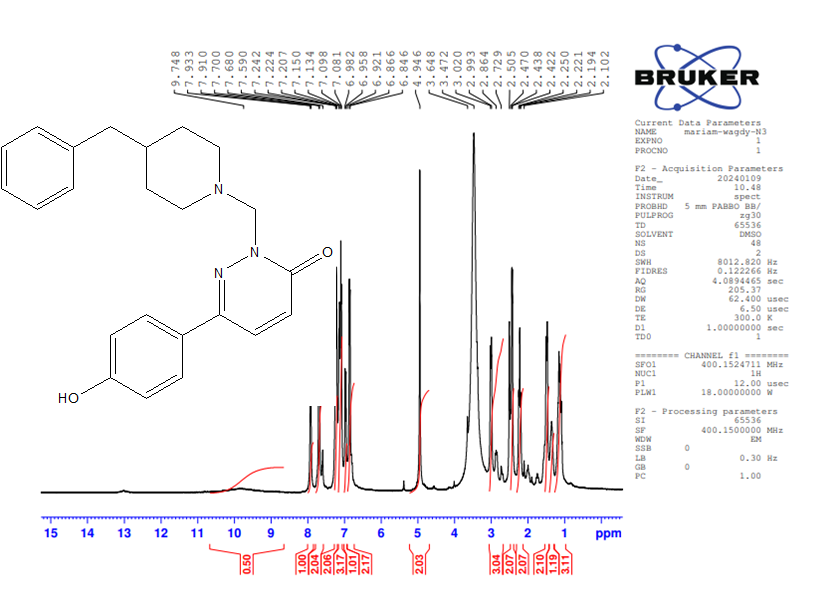
Figure S25. ^1^H NMR of 2h.


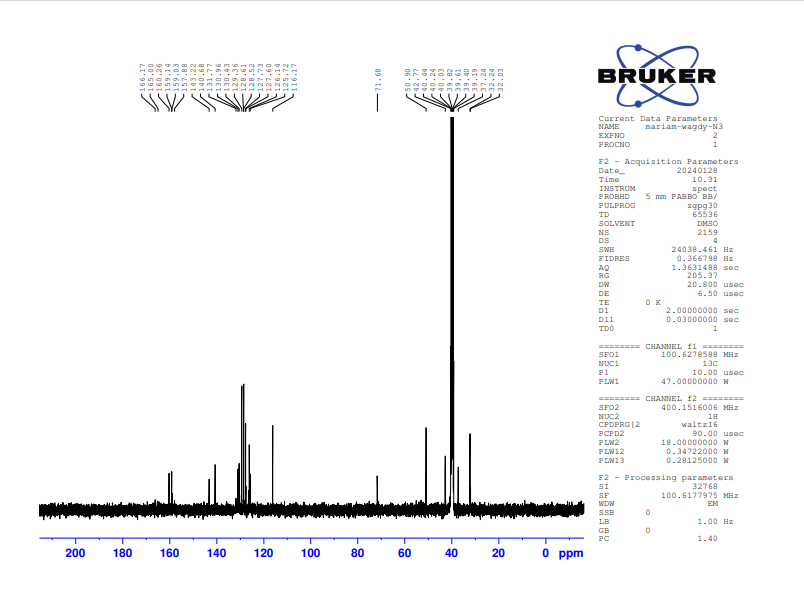


Figure S26. ^13^C NMR of 2h.


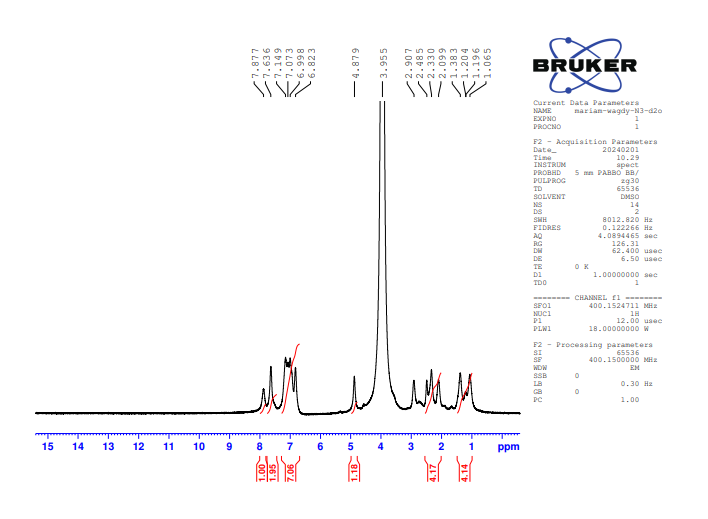
Figure S27. D_2_O of 2h.


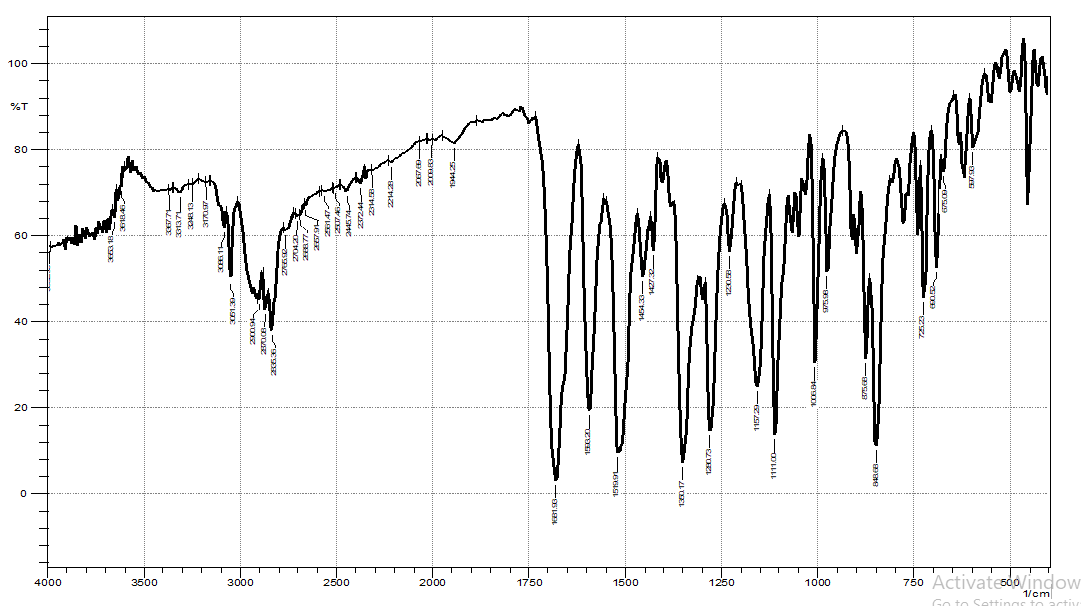
Figure S28. IR Chart of 2i.


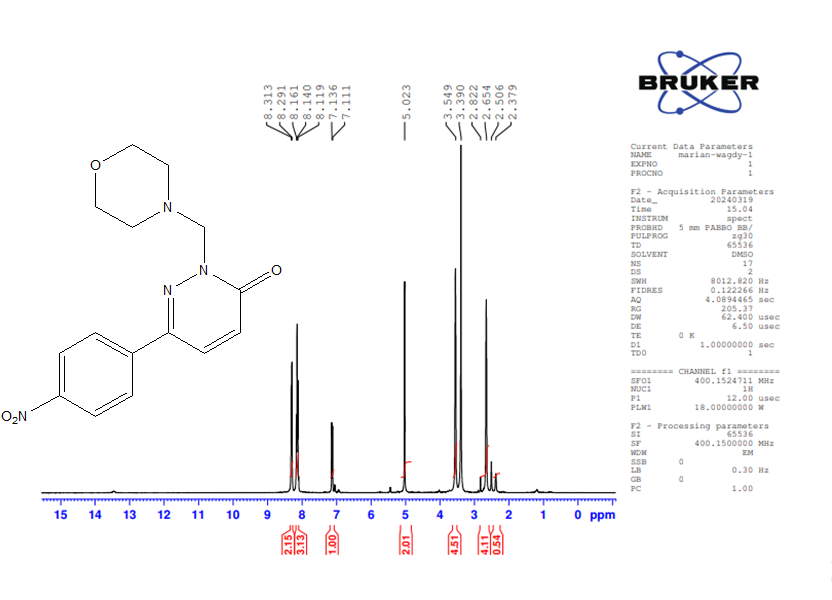
Figure S29. ^1^H NMR of 2i.


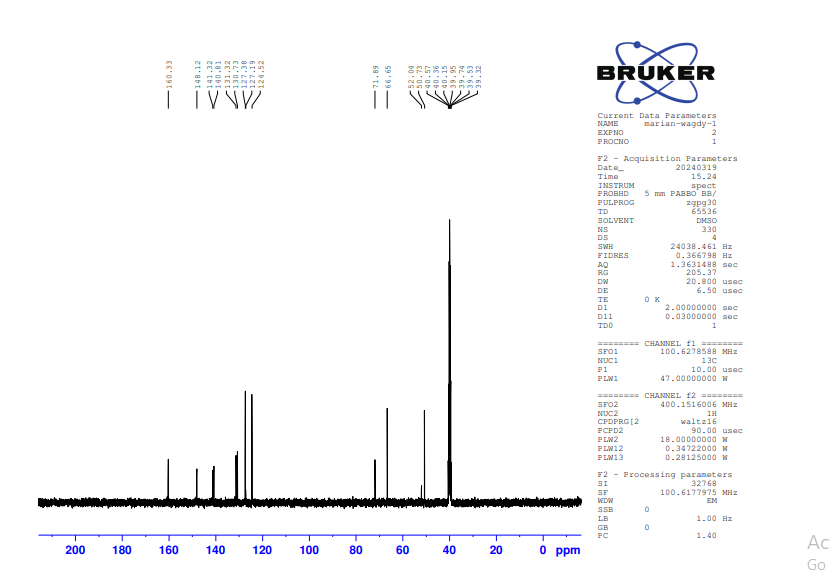
Figure S30. ^13^C NMR of 2i.


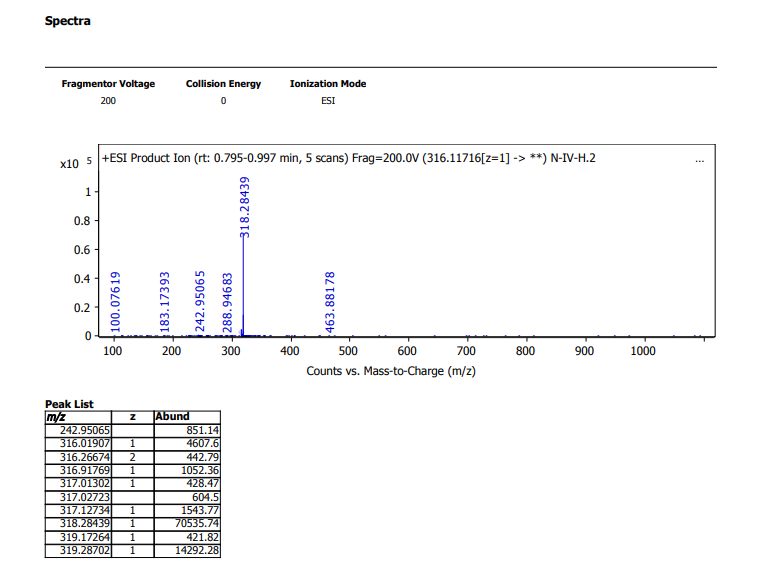


Figure S31. HR-MS Spectra of 2i.


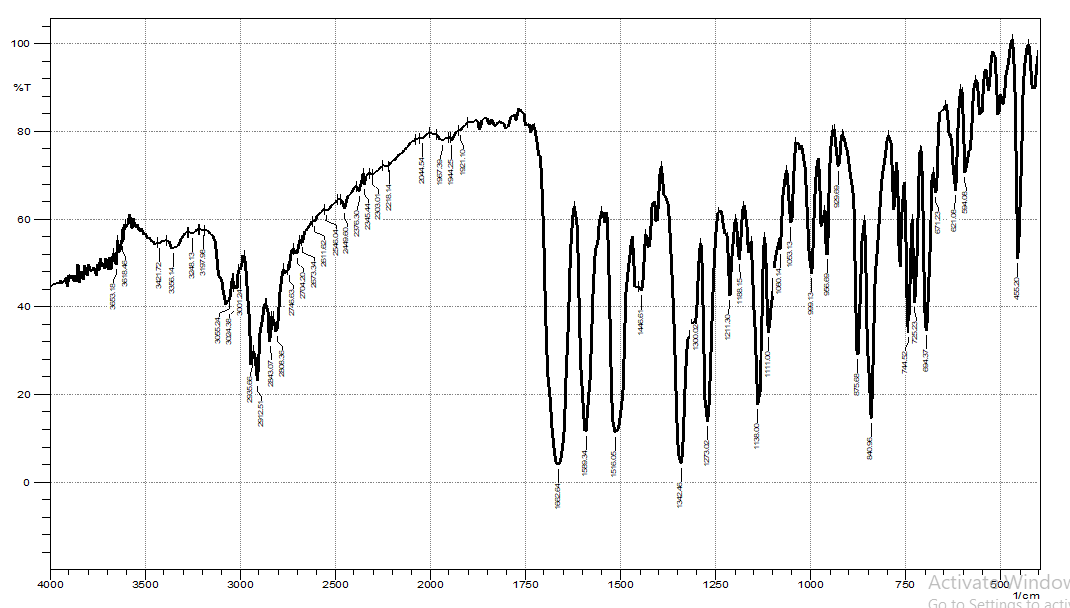
Figure S32. IR Chart of 2j.


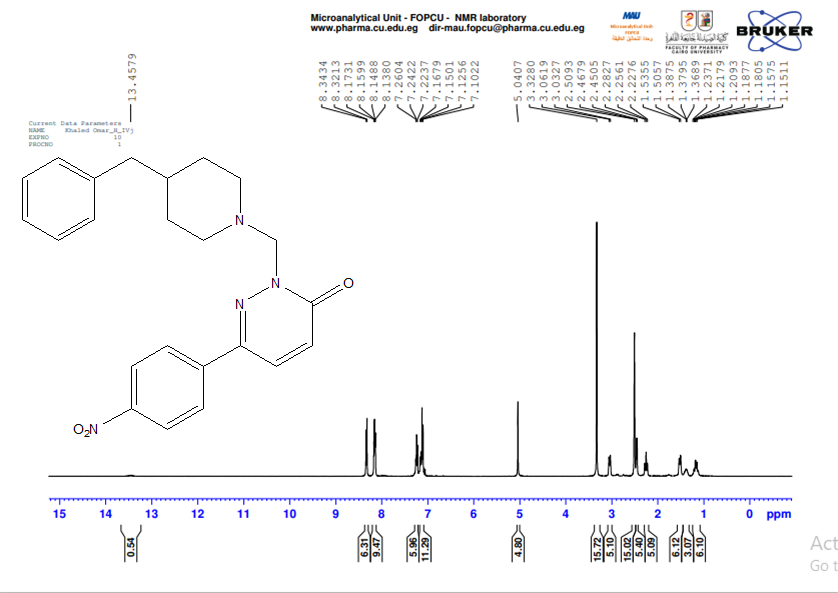
Figure S33. ^1^H NMR of 2j.


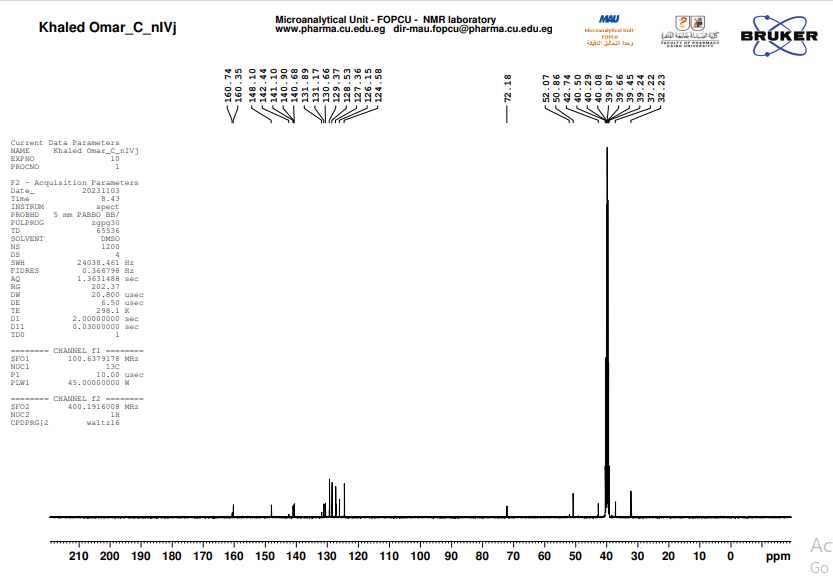
Figure S34. ^13^C NMR of 2j.
